# Isolation of BVDV-1a, 1m, and 1v strains from diarrheal calf in china and identification of its genome sequence and cattle virulence

**DOI:** 10.3389/fvets.2022.1008107

**Published:** 2022-11-16

**Authors:** Jie Zhu, Chen Wang, Lina Zhang, Tingting Zhu, Hanxiong Li, Yunqiu Wang, Kaili Xue, Mingpu Qi, Qingjie Peng, Yingyu Chen, Changmin Hu, Xi Chen, Jianguo Chen, Huanchun Chen, Aizhen Guo

**Affiliations:** ^1^State Key Laboratory of Agricultural Microbiology, Huazhong Agricultural University, Wuhan, China; ^2^College of Veterinary Medicine, Huazhong Agricultural University, Wuhan, China; ^3^Hubei Hongshan Laboratory, Wuhan, China; ^4^Hubei International Scientific and Technological Cooperation Base of Veterinary Epidemiology, Key Laboratory of Preventive Veterinary Medicine in Hubei Province, Wuhan, China; ^5^Key Laboratory of Ruminant Bio-Products of Ministry of Agriculture and Rural Affairs, Huazhong Agriculture University, Wuhan, China; ^6^Key Laboratory of Development of Veterinary Diagnostic Products, Ministry of Agriculture, Huazhong Agricultural University, Wuhan, China; ^7^Wuhan Keqian Biology Co., Ltd., Wuhan, China

**Keywords:** bovine viral virus diarrhea (BVDV), novel genotype, BVDV-1v, calf diarrhea, genetic variation, pathogenicity

## Abstract

Bovine viral diarrhea virus (BVDV) is an important livestock viral pathogen responsible for causing significant economic losses. The emerging and novel BVDV isolates are clinically and biologically important, as there are highly antigenic diverse and pathogenic differences among BVDV genotypes. However, no study has yet compared the virulence of predominant genotype isolates (BVDV-1a, 1b, and 1m) in China and the emerging genotype isolate BVDV-1v. The serological relationship among these genotypes has not yet been described. In this study, we isolated three BVDV isolates from calves with severe diarrhea, characterized as BVDV-1a, 1m, and novel 1v, based on multiple genomic regions [including 5-untranslated region (5′-UTR), Npro, and E2] and the phylogenetic analysis of nearly complete genomes. For the novel genotype, genetic variation analysis of the E2 protein of the BVDV-1v HB-03 strain indicates multiple amino acid mutation sites, including potential host cell-binding sites and neutralizing epitopes. Recombination analysis of the BVDV-1v HB-03 strain hinted at the possible occurrence of cross-genotypes (among 1m, 1o, and 1q) and cross-geographical region transmission events. To compare the pathogenic characters and virulence among these BVDV-1 genotypes, newborn calves uninfected with common pathogens were infected intranasally with BVDV isolates. The calves infected with the three genotype isolates show different symptom severities (diarrhea, fever, slowing weight gain, virus shedding, leukopenia, viremia, and immune-related tissue damage). In addition, these infected calves also showed bovine respiratory disease complexes (BRDCs), such as nasal discharge, coughing, abnormal breathing, and lung damage. Based on assessing different parameters, BVDV-1m HB-01 is identified as a highly virulent strain, and BVDV-1a HN-03 and BVDV-1v HB-03 are both identified as moderately virulent strains. Furthermore, the cross-neutralization test demonstrated the antigenic diversity among these Chinese genotypes (1a, 1m, and 1v). Our findings illustrated the genetic evolution characteristics of the emerging genotype and the pathogenic mechanism and antigenic diversity of different genotype strains, These findings also provided an excellent vaccine candidate strain and a suitable BVDV challenge strain for the comprehensive prevention and control of BVDV.

## Introduction

Bovine viral diarrhea virus (BVDV), the pathogen of bovine viral diarrhea (BVD), is an important causative agent that seriously endangers the health of livestock (especially cattle) and is a non-negligible risk factor in the life of wild ruminants (1). The virus can lead to various symptoms and clinical features, including fever, diarrhea, leucopenia, reduction in milk production and reproductive capacity, and even immunosuppression and secondary infection (2). Based on their cytopathic effect (CPE) on tissue culture epithelial cells, the BVDVs are classified into two phenotypic biotypes: cytopathic (CP) and non-cytopathic (NCP), and the latter is the most frequent form of BVD in natural infection (3, 4). According to the duration of viremia, BVDV infection can be distinguished into two types: transient infection (TI) and persistent infection (PI) (5). Persistently infected animals are viremic (virus-positive and antibody-negative or seronegative) and continuously shed large amounts of BVDV in all bodily secretions including nasal discharge, saliva, semen, and feces. PI calves are the major reservoir of viral spread within the cattle population, and BVDV is transmitted through most organs in the animal without clinical symptoms. Therefore, it is important to identify and remove these animals from the herd (6). Persistently infected cattle are a source of infection for other animals in a herd (7). BVDV is a single-strand RNA virus belonging to the genus *Pestivirus* of the family *Flaviviridae* along with other Pestiviruses such as the Classical swine fever virus (CSFV) and Border disease virus (BDV) (4). The genome is an ~12.3 kb positive-stranded RNA (8). Its 5-untranslated region (5′-UTR), a single open reading frame (ORF), and the 3-untranslated region (3′-UTR) make up the BVDV genome. The ORF encodes 12 proteins, including four structural proteins (capsid, Erns, E1, and E2) and eight non-structural proteins (N^pro^, p7, NS2, NS3, NS4A, NS4B, NS5A, and NS5B) (9, 10).

Based on the phylogenetic analysis of partial and complete genomic sequences, BVDVs are divided into two genotypes: BVDV-1 and BVDV-2 (11). Previous studies have reported that BVDVs are highly genetically diverse, even within the individual genotypes (11). BVDV-1 and 2 are further divided into multiple genetic genotypes 1a-1u and 2a-2d (11, 12). Partial sequences of the 5′-UTR are most frequently used for phylogenetic analysis and genotyping of BVDV, followed by the N^pro^ and E2 coding sequences. However, the determination of BVDV phylogenies and classification based on 5′-UTR alone is limited (13, 14). The main disadvantages are the limited sequence length and the lack of genetic diversity (11). One of the typical examples is the identification of the recent novel genotype BVDV-1v from a Chinese cattle herd (15). Due to the lack of a complete genome sequence for the joint analysis of multiple gene regions, some isolates identified as BVDV-1v based on 5′-UTR are classified as BVDV-1o based on N^pro^ (16, 17). Thus, using only the 5′-UTR or Npro region is not the best option for a comprehensive and detailed phylogenetic analysis. To define species or genotypes, the examination of multiple BVDV genomic regions is indispensable to concluding the phylogenetic relationship between BVDV strains (18). Therefore, a complete genome sequencing of the novel genotype BVDV-1v isolates is particularly important for accurately classifying this novel genotype. In addition, the different BVDV genotypes originate from a common ancestor and spread in cattle that accompanied human activity in different geographic regions (19). The complete genome sequences contribute to determining the origin of the BVDV-1 genotype's genetic variation, especially the emerging genotypes.

Viral virulence is significant to understanding the pathological mechanisms and selecting the challenging strains to evaluate vaccines. The virulence differences among BVDV-2 strains have been reported (20), but much less information is available on the virulence changes among BVDV-1 different genotype strains. Currently, BVDV-1a, 1b, and 1m are the major prevalent genotypes in China (12, 15), and BVDV-1v, an emerging genotype in China, is also a regional prevalence (15–17). However, there is no description of the pathogenicity of BVDV-1m and 1v isolates to natural host cattle, which limits our understanding of the pathogenic mechanism of dominant BVDV-1 strains in China. In addition, the high genetic diversity among different BVDV genotypes also generally reflects their antigenic difference, which is significant for disease prevention and vaccination programs (21). Previous studies showed that antigenic significant differences and similarities are observed between the BVDV-1 genotypes (21, 22). Although BVDV-1a-inactivated vaccines have recently been used to prevent the spread of BVDV in China, the serological cross-reactivities of 1a with 1m and 1v remain unclear.

Considering the above scientific gaps, it is necessary to isolate these BVDV genotypes, obtain complete genome sequences, and perform animal infection experiments on these isolated genotypes. In this study, we successfully isolated three BVDV isolates from calves with severe diarrhea. The phylogenetic analysis based on multiple gene regions (including 5′-UTR, N^pro^, and E2) and nearly complete genome indicated that the three isolates (HN-03, HB-01, and HB-03) are BVDV-1a, 1m, and novel genotype 1v, respectively. For the emerging genotype BVDV-1v, the evolutionary genetic characteristics and potential evolution origin were determined based on the complete genome sequence. Furthermore, we evaluated the pathogenicity of the three BVDV isolates to newborn calves and determined the serological relationship among these genotypes. These findings will help with efforts to develop control measures against BVDV.

## Materials and methods

### Sample collection, cells, and viral strains

From March to December 2017, three cattle farms from Hubei province (*n* = 2) and Henan Province (*n* = 1), China, had cases of severe diarrhea among newborn calves with respiratory symptoms. All diarrheic calves ranged from 1 to 6 weeks old and had not been vaccinated with the BVDV vaccine. Blood was collected from the jugular of a diarrheic calf, and serum was isolated, kept in cold storage at about 4°C, and immediately transported to the laboratory, where they were stored at −80°C until use.

MDBK (Madin-Darby bovine kidney) cells (ATCC CCL-22) and the BVDV AV69 (BVDV-1b, GenBank: KC695814) strain were procured from our laboratory stocks.

### Viral detection

All serum samples were screened for antigen (Ag) to BVDV using commercial BVDV enzyme-linked immunosorbent assay (ELISA) kits (IDEXX Laboratories, Inc., USA). All Ag-positive samples were also tested with reverse transcription-polymerase chain reaction (RT-PCR) for the 5′-UTR and N^pro^ region of BVDV, following the protocol previously reported (12).

### Viral isolation and characterization

To isolate the virus, the 200 μL BVDV-positive serum samples were inoculated into MDBK cells following the protocol previously reported (23). To avoid potential BVDV antibodies in commercial bovine serum, we used horse serum (Gibco, USA), instead of bovine serum for cell culture. To identify the isolated BVDV strains, the supernatant after the third passage was identified by immunofluorescence assay (IFA) according to the methodology described in a previous study (23). The subtle differences in the IFA method were that the MDBK cells were successively incubated with BVDV-1&2 MAb (VMRD, USA) and Donkey Anti-Mouse IgG(H + L) (Invitrogen, USA), respectively.

Viruses were diluted in a series of tubes 10 times and their presence identified by the IFA method in place of cytopathic effects (CPEs). The viral titer was measured by median tissue culture infectious dose (TCID_50_) based on the Reed–Muench method (24). We further determined the one-step growth curve of the three isolates BVDV-1a HN-03, BVDV-1m HB-01, and BVDV-1v HB-03. The monolayer MDBK cells in a 6-well culture plate (Corning, USA) were infected with the three BVDV isolates with 5 TCID_50_/cell and incubated for 1 h at 37°C. Then, removing the inoculum and rinsing the cells 3 times with pre-warmed Dulbecco's modified Eagle's medium (DMEM) (Gibco, USA), the monolayer cells per well were supplemented with 2 mL DMEM (10% horse serum) and further cultivated at 37°C. The TCID_50_ of the three BVDV isolates were titrated at 0, 3, 6, 9, 12, 15, 18, 21, 24, 30, 36, 48, and 72 h post-infection (hpi).

### Genome and phylogenetic analysis

Three BVDV strains in passage cultures were used for complete genome sequencing based on the next-generation sequencing technology (25). In addition, one pair of primers (Supplementary Table 1) was designed to amplify the complete genome sequence of the BVDV-1v HB-03 strain following the protocol reported previously (25). The RT-PCR products were cloned into the pMD19-T vector and sequenced in Tsingke Biotech (Beijing, China). These three BVDV genome sequences were submitted to the GenBank with the accession numbers ON901783–ON901785.

ORF finder (https://www.ncbi.nlm.nih.gov/orffinder/) was used to search for ORFs in the three BVDV isolate sequences obtained in this study. Neighbor-joining analysis of the nucleotide sequences in different genomic regions of BVDV strains was performed using MEGA 7 (Molecular Evolutionary Genetics Analysis). The unrooted phylogenetic trees were constructed with bootstrap values calculated for 1,000 replicates (26). The nucleotide sequence identities of the three BVDV isolates with 25 representative strains were calculated using the ClustalW algorithm in DNAstar MegAlign software. Similarity plots and boot scanning analyses were performed using the SimPlot software package (version 3.5.1) with the default parameter settings (27). Bootscan analysis was performed using the neighbor-joining tree model and the Kimura 2-parameter distance algorithm with a window of 200 bp and a step of 20 bp. The recombination events in BVDV-1v HB-03 isolate complete genome were further determined using the Recombinant Detection Program (RDP4, v4.46) with seven methods (RDP, GENECONV, MaxChi, Bootscan, Chimera, SiScan, and 3Seq) (28). Genomes with significant evidence (*p* < 0.05) of a recombination event obtained with at least four methods were indicated. The E2 of BVDV-1v HB-03 isolate in the tertiary structure was predicted using I-TASSER (https://swissmodel.expasy.org/interactive), and the surface representations of the tertiary structure were generated with the PyMOL (http://www.pymol.org/). The information on reference strains is shown in Supplementary Table 2, including the strains, genotype, origin, location, collection time, and GenBank accession numbers.

### Experimental infection of animals

#### Animal selection

Twelve healthy Holstein weaned calves (mean age 52.3 ± 4.5 days old and mean weight 62.4 ± 6.7 kg) were selected from a dairy farm in Henan province, China. The criteria include: the animals were free from antigens to BVDV in calves' blood using ELISA kits (Idexx Laboratories, Inc., USA), infectious bovine rhinotracheitis virus in calves' nasal swabs (NSs) based on viral isolation and PCR (29), and *Mycoplasma bovis* in calves' nasal swabs based on isolation and PCR (30), and antibodies of BVDV tested by using ELISA kits (Idexx Laboratories Inc., USA) for animal serums. In addition, all animals' fecal samples were also confirmed to be negative for the bovine enteric pathogens (*Escherichia coli* F5, bovine rotavirus, bovine coronavirus, and *Cryptosporidium parvum*) using commercial antigen detection ELISA kits (IDEXX Laboratories, Inc., USA). The selected animals were transported to our experimental farm in Songzi city, Hubei province.

#### Virus inoculation

**Figure 1:**
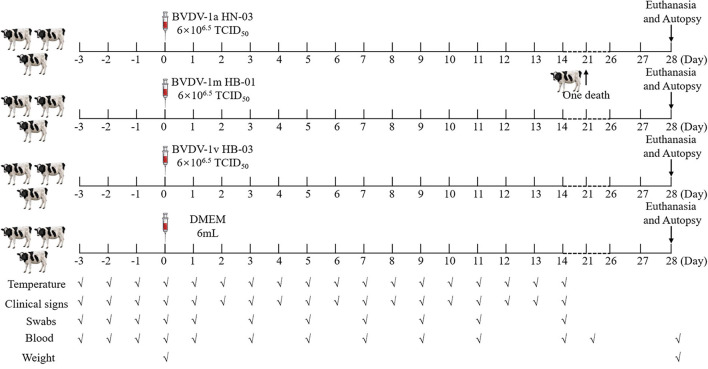
The animal experiment protocol. Calves were intranasally infected with bovine viral diarrhea virus (BVDV). All animals were euthanized and necropsied at 28 dpi, except for one animal (BVDV-1a HN-03 group) that died at 21 dpi. The markers below indicate the measures and sample collection on the day.

The mean rectal temperature of all animals was measured twice per day (at 7 am and 5 pm) and for three continuous days before viral infection. The mean rectal temperature of all calves ranged within ±0.5°C during these 3 days, confirming they were clinically healthy. Then, 12 calves were randomly assigned to four groups, three infection groups (inoculated with strains BVDV-1a HN-03, BVDV-1m HB-01, or BVDV-1v HB-03), and one negative control group. The calves from three infection groups were infected with 6 mL of a viral culture supernatant containing 10^6.5^ TCID_50_/mL of the different BVDV strains, while the calves from the negative control group were mock-infected with 6 mL DMEM. The inoculum (virus or DMEM) was instilled into the nostrils of calves, 3 mL for each nostril. All animals were euthanized and necropsied at 28 dpi (days post-infection), except for one animal (BVDV-1a HN-03 group) that died at 21 dpi, and their tissues were aseptically collected. The calf infection program is shown in Figure 1.

#### Clinical signs and sample collection

The clinical signs were observed and scored for all calves from 1 to 14 days post-infection (dpi) (Supplementary Table 3). The clinical signs of all calves were recorded, including conjunctivitis, nasal discharge, coughing, abnormal breathing, diarrhea, and appetite. Scores ranged from 0 to 3, with higher scores indicating more severe symptoms. The body weights of all calves were measured before starting and after termination of the observation. Blood, nasal swabs (NSs), and rectal swabs (RSs) samples were immediately transported to the laboratory in cold storage at about 4°C. Blood samples were collected in EDTA (ethylenediaminetetraacetic acid) tubes at the date of examination, and white blood cell (WBC) and lymphocyte (LYM) counts were measured on fresh samples shortly using the VetscanHM5 veterinary hematology system (Abaxis, USA).

#### Virus shedding test

Viruses were isolated from NSs, RSs, and WBC, as described above. The viral load in samples (NSs, RSs, and blood) was determined by reverse transcription quantitative polymerase chain reaction (RT-qPCR) based on specific primers (F:5-CATGCCCWYAGTAGGACTAGC-3, R:5-TCCATGTGCCATGTACARCAGAG-3). All samples were preliminarily processed, and then the viral RNA was extracted with a QIAamp Viral RNA/DNA Mini Kit (QIAGEN, Germany). Reverse transcription (RT) was performed using the reagents of the PrimeScript II Reverse Transcriptase (Takara, Otsu, Shiga, Japan), following the manufacturer's protocol. The complementary DNA (cDNA) was obtained from these samples as per the above description. qPCRs were performed using AceQ qPCR SYBR Green Master Mix (Vazyme, China) and run in triplicate using an ABI 7500 thermocycler (Applied Biosystems, CA, USA), and the data were analyzed using model 7500 SDS software v 1.3.1.

#### Systemic distribution of viral RNA

To determine the systemic distribution of BVDV in inoculated calves, viral RNAs were determined by RT-PCR in different tissues from euthanized calves at 28 dpi. Viral RNAs of calves' organs (hearts, livers, spleens, lungs, kidneys, small intestines, tonsils, lymph nodes, and serums) were extracted using the commercial RNAprep Pure Tissue Kit (TIANGEN, China). The cDNA was obtained from these samples given in the above description. All organ samples were tested with RT-PCR for 5′-UTR of BVDV following the protocol previously reported (12).

#### Histopathological study

All animals were euthanized and necropsied at 28 dpi, except for one animal (BVDV-1a HN-03 group) that died on the 21 dpi, and their tissues (spleens, lungs, and intestines) were aseptically collected. The samples were immediately put in 10% neutral buffered formalin solution for fixation and kept for histopathological examination.

#### Test of neutralizing antibodies

Virus neutralizing antibodies of the calves infected with BVDVs were determined in MDBK cells grown in 96-well microplates as given in the previous reports (31, 32). The presence of each BVDV isolate after being neutralized by the serums was determined by the IFA method, as described above. Serum titers of neutralization antibodies were expressed as the reciprocal of the highest serum dilution that neutralized the 100 TCID_50_ of the virus suspension. To characterize the antigenic relatedness among BVDV genotypes, the coefficient of antigenic similarity was calculated based on the formula given previously (21). By this formula, a ratio (*R*) equal to or close to 100 was determined to have no significant antigenic difference.

### Statistical analyses

All statistical tests of the data were performed using GraphPad Prism software (version 8.0). The data were shown as mean ± SD of three independent or three replicate samples. The statistical significance was calculated using Student's *t*-test for one comparison and analysis of variance (ANOVA) for multiple comparisons. *P* < 0.05 was considered to show statistically significant differences.

## Results

### Viral isolation, identification, titer, and one-step growth curve

**Figure 2:**
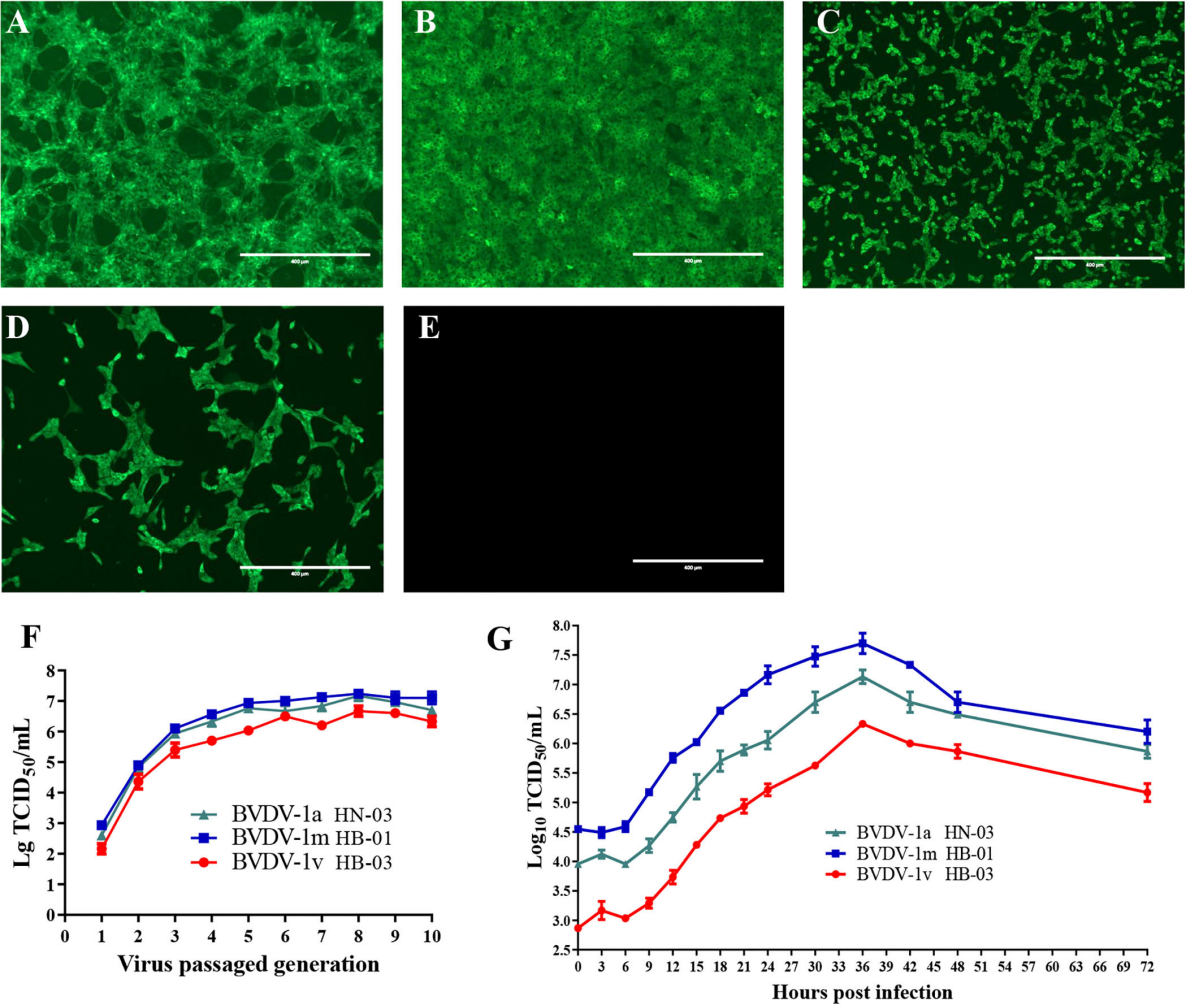
Viral isolation and characterization. The specific immunofluorescent signals were detected in the cytoplasm of cells inoculated with BVDV-1a HN-03 **(A)**, BVDV-1m HB-01 **(B)**, and BVDV-1v HB-03 **(C)**. Madin-Darby bovine kidney (MDBK) cells inoculated with BVDV-1b AV-69 (as a positive control) cells showed specific fluorescence **(D)**, and mock-infected cells showed no bright fluorescence **(E)**. Median tissue culture infectious dose (TCID_50_) of the three bovine viral diarrhea virus (BVDV) isolates inoculated with MDBK in passages **(F)**. One-step growth curves of BVDV-1a HN-03, BVDV-1m HB-01, and BVDV-1v HB-03 isolates **(G)**.

To isolate the BVDVs, three BVDV-positive serum samples of the diarrheal calves from three different farms were inoculated into MDBK cells. After three passages, no obvious CPEs were observed in the three inoculated MDBK cell wells. However, the specific immunofluorescent signals were both detected in the cytoplasm of three inoculated cells using BVDV-1&2 Mab (Figures 2A–C). MDBK cells inoculated with BVDV-1b AV-69 (as a positive control) showed specific fluorescence (Figure 2D), and mock-infected cells showed no bright fluorescence (Figure 2E). To assure the above results, RT-PCR was performed and fragments were obtained from inoculated cells, and revealed they were indeed BVDV-1-specific sequences. The three BVDV isolates isolated from diarrheal calves were designated as BVDV-1/Bovine/CHN/HN-03/2017 (abbreviated BVDV-1a HN-03), BVDV-1/Bovine/CHN/HB-01/2017 (abbreviated BVDV-1m HB-01), and BVDV-1/Bovine/CHN/HB-03/2017 (abbreviated BVDV-1v HB-03), respectively, and used for further characterization.

The titer of the three BVDV isolates increased rapidly with the increase of passages before the fifth passage, and finally stabilized at TCID_50_ = 10^−(6.0−7.5)^/mL (Figure 2F). To compare the replication levels of the three BVDV isolates, one-step growth curves were performed after inoculation using the same amount of BVDV (5 TCID_50_/cell). As shown in Figure 2G, all BVDV isolates exhibited a similar growth trend, and at 36 hpi, replication of these viruses reached the peak level. The TCID_50_ values of the BVDV-1v HB-03 strain at each time point were lower than those of the BVDV-1m HB-01 strain and BVDV-1a HN-03 strain.

### Genomic characterization and evolution analysis of three BVDV isolates

#### Genomic information and phylogenetic analysis

**Figure 3:**
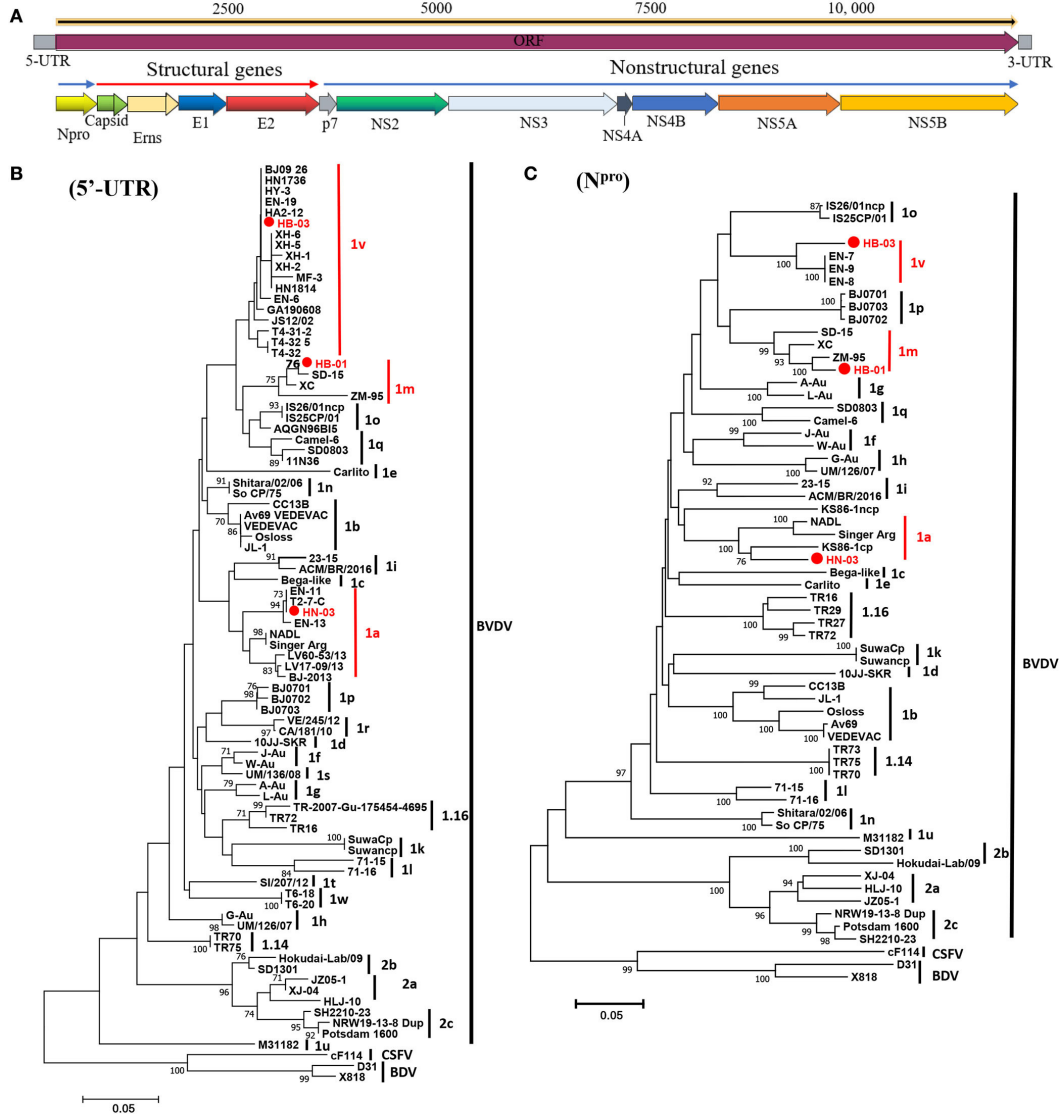
Schematic diagram of the genomic structure and phylogenetic analysis. Schematic diagram of the annotated bovine viral diarrhea virus (BVDV) genome **(A)**. Phylogenetic tree of the nucleotide sequences of the 5-untranslated region (5′-UTR) **(B)** and N^pro^ **(C)**. Molecular evolutionary genetics analysis was performed with MEGA 7 (Molecular Evolutionary Genetics Analysis) using the neighbor-joining (NJ) and bootstrap analysis (*n* = 1,000) method. Unrooted trees described the relationship between sequences retrieved pestivirus [BVDV-1, BVDV-2, classical swine fever virus (CSFV), and border disease virus (BDV)] from the GenBank database and the three BVDV isolates analyzed in this work (red circle). Information on the reference strains is shown in Supplementary Table 3.

**Figure 4:**
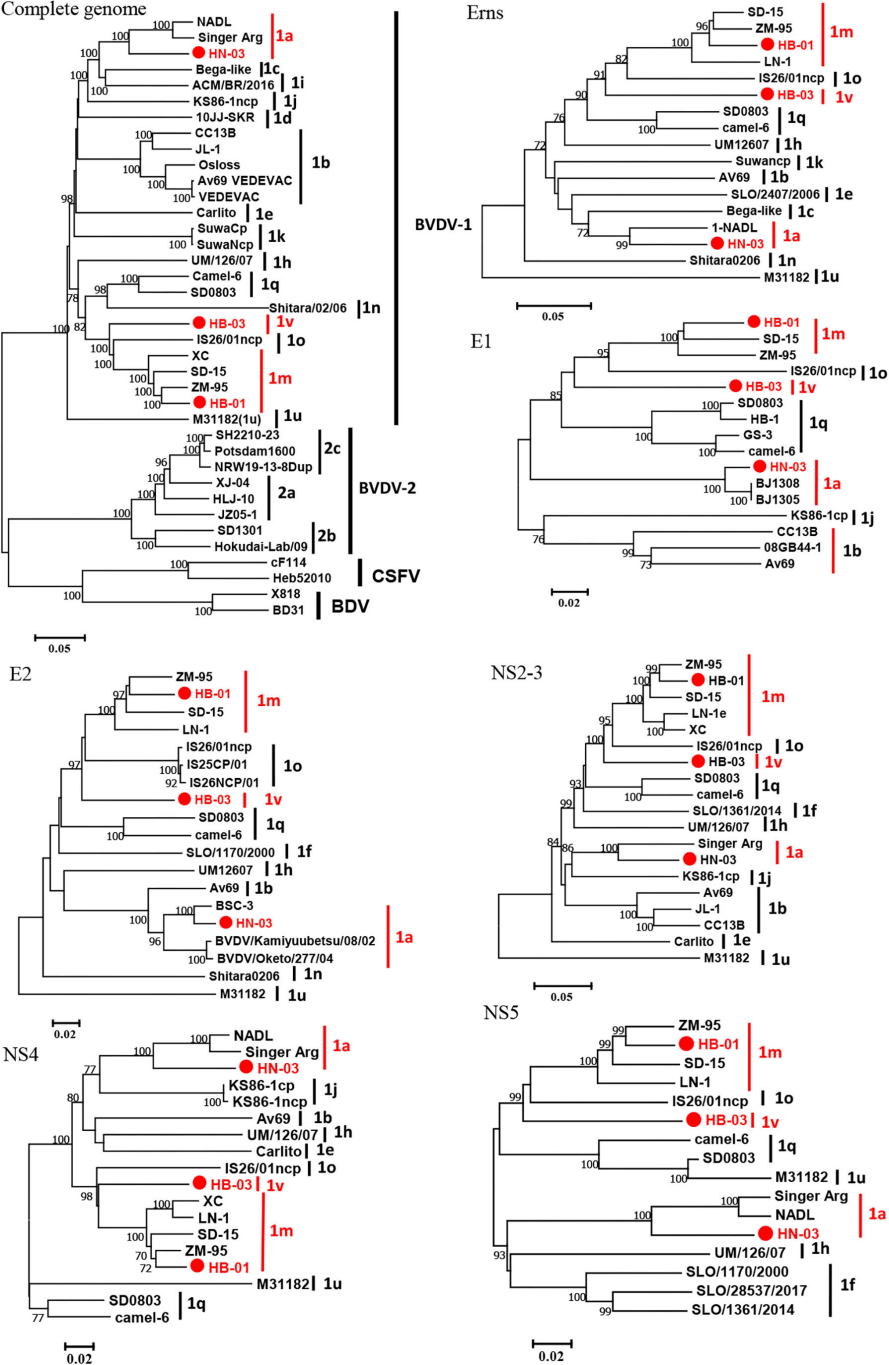
Phylogenetic tree of the nucleotide sequences of the complete gene and six segments (Erns, E1, E2, NS2-3, NS4, and NS5). Molecular evolutionary genetics analysis was performed with MEGA 7 (Molecular Evolutionary Genetics Analysis) using the neighbor-joining (NJ) and bootstrap analysis (*n* = 1,000) method. Unrooted trees described the relationship between sequences retrieved from the GenBank database and the three bovine viral diarrhea virus (BVDV) isolates analyzed in this work (red circle). Information on the reference strains is shown in Supplementary Table 3.

The complete genome lengths of BVDV-1a HN-03, BVDV-1m HB-01, and BVDV-1v HB-03 strains were 12,307, 12,189, and 12,155 nucleotides (nt), respectively. They possessed the same genome structure (Figure 3A), including 5′-UTR of 267-382 nt, 3′-UTR of 157–238 nt, and an open reading frame (ORF) encoding a large precursor polyprotein of 3,898–3,900 amino acids (aa) (Supplementary Table 4). To identify the genotype of the three isolates, the phylogenetic tree 5′-UTR was constructed using the neighbor-joining method. Phylogenetic analysis indicated that the HN-03 and HB-01 strains were identified as BVDV-1a and BVDV-1m, respectively (Figure 3B). The BVDV-1v HB-03 strain formed a subcluster together with Chinese BVDV strains (Figure 3B), and this subcluster has been identified as a novel genotype of BVDV-1v (15). Furthermore, the N^pro^ phylogenetic tree analysis confirmed the above identification of BVDV-1a, BVDV-1m, and the novel genotype BVDV-1v (Figure 3C). In addition, based on the phylogenetic analysis of the complete genome and six segments (Erns, E1, E2, NS2-3, NS4, and NS5), the BVDV-1v HB-03 strain was also clustered in a separate branch (Figure 4), indicating the reliability in identifying the novel genotype BVDV-1v.

**Figure 5:**
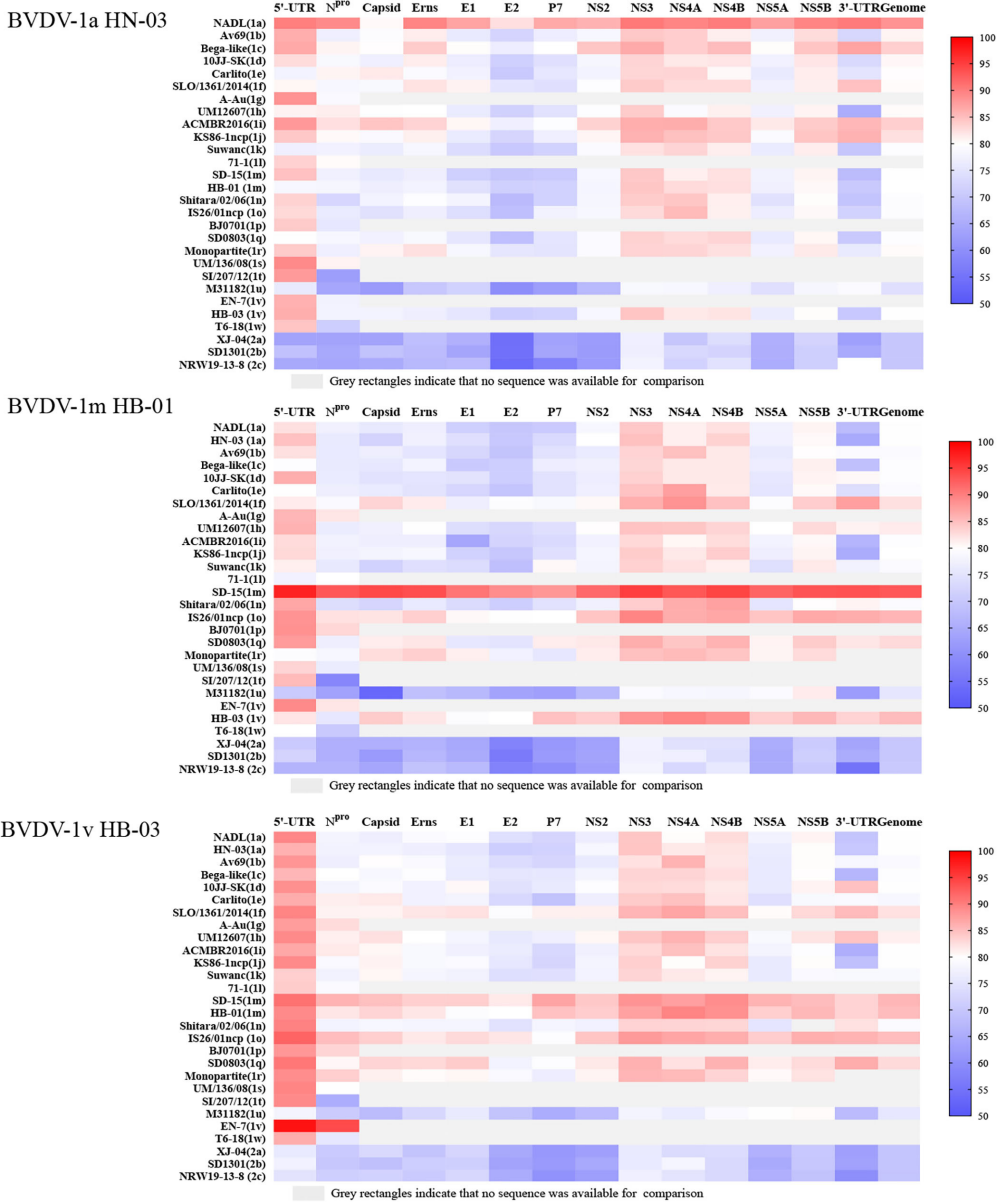
Nucleotide sequence identity (%) comparison of each segment of the three bovine viral diarrhea virus (BVDV) isolates to other strains of BVDV-1 and 2. Information on the reference strains is shown in Supplementary Table 3.

To determine the nucleotide identity between the three BVDV strains in this study and other genotype stains, the complete genome and multiple gene regions' (5′-UTR, N^pro^, Capsid, Erns, E1, E2, p7, NS2, NS3, NS4A, NS4B, NS5A, NS5B, and 3′-UTR) sequences of the three isolates in this study were aligned against corresponding sequences of the BVDV 1a-1u and 2a-2c strains. The BVDV-1a HN-03 and BVDV-1m HB-01 strains were most closely related to Chinese strains BVDV-1a GS5 (91.6%) (33) and BVDV-1m SD-15 (93.1%) (23), respectively. Similarly, the two isolates (BVDV-1a HN-03 and BVDV-1m HB-01) in this study possessed the highest identity with their corresponding genotype strains in each gene region (Figure 5). Although the novel genotype BVDV-1v has been reported in several studies (15, 16), there is currently no information about the complete genome sequence of this genotype. Therefore, the complete genomic identity between the BVDV-1v HB-03 strain and the retrieved BVDVs in GenBank (as of August 31, 2021) was <90%, ranging from 75.6 to 85.7% and from 69.5 to 69.6% with the BVDV-1 and BVDV-2 strains, respectively (Figure 5). The BVDV-1v HB-03 strain had the highest nucleotide identity of 85.7% with the BVDV-1m SD-15 strain, a prevalent dominant genotype in China originally isolated from bovine showing pyrexia, oral mucous ulcer, and severe diarrhea in Shandong province, China in 2015 (23). In addition, the BVDV-1v HB-03 strain also showed a high nucleotide identity in the complete genome sequence to the BVDV-1m HB-01 strain (85.2%), following with bovine strain BVDV-1o IS26/01ncp from Japan (85.1%), and porcine strain BVDV-1q SD0803 from China (82.9%), indicating the close genetic relationship between BVDV-1v and these genotypes (1m, 1o, and 1q).

#### Recombination analysis

**Figure 6:**
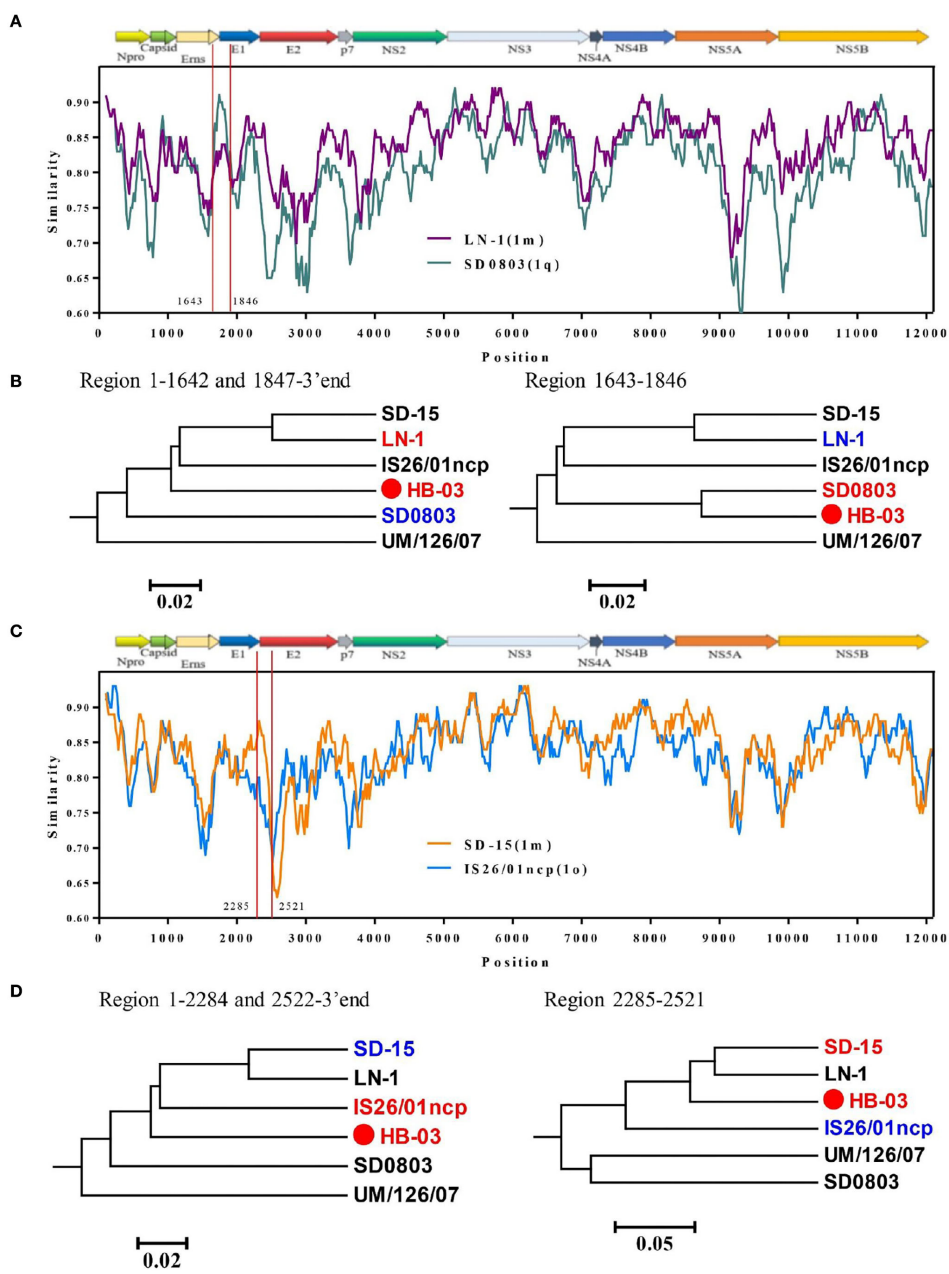
Recombination analysis of the BVDV-1v HB-03 strain genome. Similarity plots and boot scanning analyses were performed using the SimPlot (Similarity Plotting) software package (version 3.5.1). Recombination breakpoints are shown with red lines, and the locations are shown at the side. **(A,C)** Show that there were two potential recombination events in BVDV-1v HB-03, respectively. Phylogenies of the major and minor parent regions are shown below the similarity plot **(B,D)**.

As the BVDV-1v genotype is emerging, understanding its evolutionary origin is necessary to prevent the spread of BVDV. Therefore, we performed a genetic recombination analysis on the BVDV-1v HB-03 strain. The complete genome of the BVDV-1v HB-03 strain was aligned against 33 representative strains of BVDV-1 and BVDV-2 genomes (Supplementary Table 2) by the ClustalW program in MEGA 7 for similarity plotting (Figure 6) and recombination analyses (Table 1). The similarity plot and RDP 4 program analysis indicated that the BVDV-1v HB-03 strain was observed with significant evidence (at least four methods in the RDP 4 program) of two recombination events. One recombination event was identified in the BVDV-1v HB-03 strain, and its parental strains were identified as BVDV-1m (LN-1 strain isolated from cattle in China) and BVDV-1q (SD0803 strain isolated from a pig in China), respectively. The other recombination event revealed that the BVDV-1v HB-03 parental strains were identified as BVDV-1o (IS26/01 ncp strain isolated from cattle in Japan) and BVDV-1m (SD-15 strain isolated from cattle in China). Similarity analyses confirmed that the BVDV-1v HB-03 strain had two potential signatures of genetic exchange in the 1,643–1,846 and 2,285–2,521 nucleotides, respectively (Figures 6A,C). In addition, the two recombination events were both verified by phylogenetic analyses of the different parental regions (Figures 6B,D).

**Table 1:** RDP4 analysis of recombination events in BVDV-1v HB-03 genomes.

**Recombination**	**Location(s)** **[nt (99% CI)^a^**	**Genetic partner** **(Major parent, Minor parent)**	**Method** ^**b**^							**Consistency^c^**
			**RDP**	**Boot scan**	**MaxChi**	**Chimera**	**GeneConv**	**SiScan**	**3Seq**	
Segment 1	1,643 (1,474–1,682), 1,846 (1,702–2,003)	LN-1(1m), SD0803(1q)	–	2.934 × 10^−04^	4.790 × 10^−04^	4.094 × 10^−05^	–	1.124 × 10^−04^	–	*
Segment 2	2,285 (ND), 2,521 (ND)	IS26/01ncp(1o), SD-15(1m)	–	3.086 × 10^−02^	2.574 × 10^−03^	2.764 × 10^−04^	–	1.060 × 10^−04^	4.631 × 10^−02^	**

a The 99% confidence interval (CI) is shown as the nucleotide segment from X to Y. ND, not determined.

b The seven tests for recombination were implemented in the RDP4 program.

c Asterisks indicate the number of methods used to determine statistical evidence of a recombination event: **, 5 methods; *, 4 methods. Genomes in which statistical evidence of a recombination event was obtained with fewer than four methods are not indicated.

#### Unique mutation analyses in the E2

**Figure 7:**
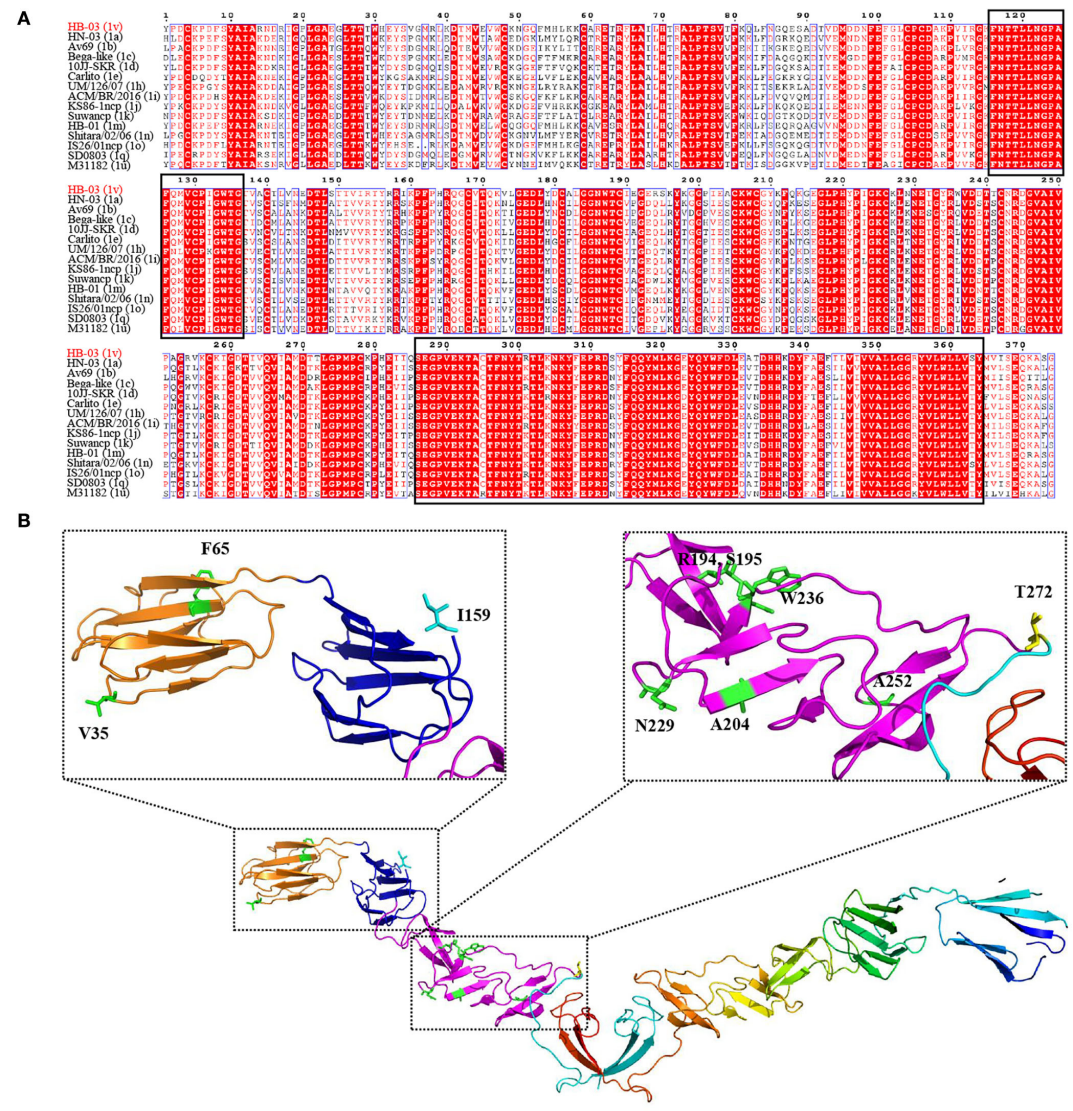
Unique mutation analyses in the E2. **(A)** Sequence alignment of BVDV E2 sequences. Two highly conserved regions within the E2 sequence were revealed to locate at the amino acid positions of 116–136 and 286–364, respectively (black rectangle). **(B)** The cartoon schemes of the BVDV-1v HB-03 strain E2 protein structure. The domains of BVDV E2 modified from El Omari et al. (35). Domain DA, comprising residues 4–87, is in orange, and domain DB, comprising residues 88–164, is in blue. Domain DC, comprising residues 165–271, is in light purple, and domain DD, comprising 272–333, is in dark fluorescent green.

Glycoprotein E2 plays an important role in immune function and viral pathogenicity, and is the main target of neutralizing antibodies (34, 35). To determine the variation of the important glycoprotein of the novel genotype, alignment analyses of E2 amino acid sequences between BVDV-1v HB-03 strain and representative BVDV-1 strains (BVDV-1a, 1b, 1c, 1d, 1e, 1h, 1i, 1j, 1k, 1m, 1n, 1o, 1q, and 1u) were performed (Figure 7). The sequence marked in red indicates a region in which the amino acid was highly conserved, and conversely, a region in which no color remarked was identified as having an amino acid difference. Two highly conserved regions within the E2 sequence were found to locate at the amino acid positions of 116–136 and 286–364, respectively (Figure 7A). However, there were 10 unique amino acid sites in E2 of the BVDV-1v HB-03 strain (Figure 7B, Supplementary Table 5). A particular amino acid site Y65F was found in antigenic domains DA (amino acids 4–87) of E2 only in the BVDV-1v HB-03 strain. Whether this particular and other different amino acid sites in the BVDV-1v HB-03 strain affect pathogenicity or antigenicity is unknown and is the subject of future investigation.

### Experimental infection of animals

#### Clinical signs

**Figure 8:**
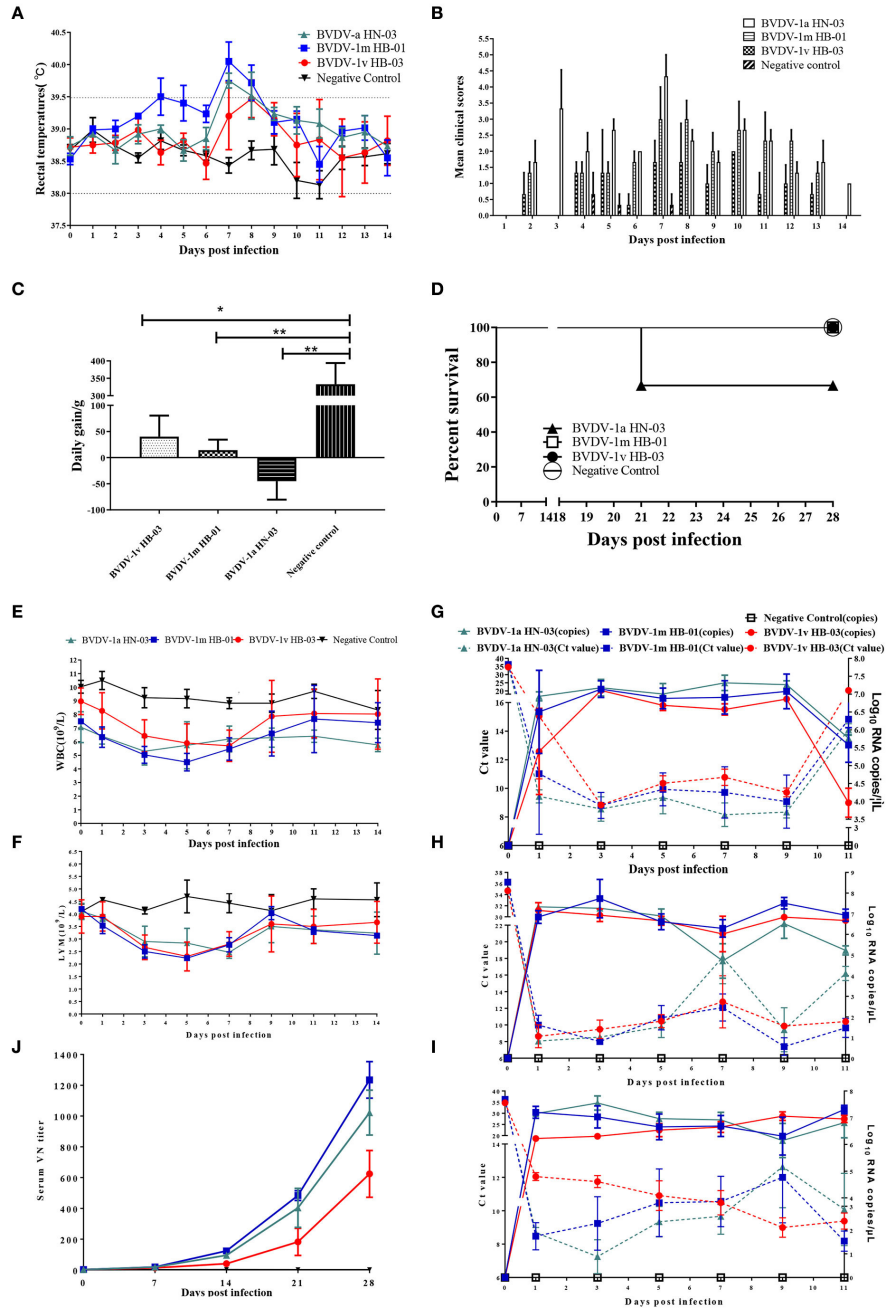
Evaluation of the three bovine viral diarrhea virus (BVDV) isolates' pathogenic and serological characters. **(A)** Fever **(B)**, clinical signs **(C)**, average daily weight gain **(D)**, and survival rate **(E)** of all calves were monitored daily. Hematology of calves infected with BVDV isolates was measured at 0–14 dpi. Mean number expressed as mean ± standard error for white blood cell (WBC) **(E)** and lymphocytes (LYM) **(F)**. Relative quantification of virus load in blood **(G)**, nasal swabs (NSs) **(H)**, and rectal swabs (RSs) **(I)** provided insights into the viral load of the virus following intranasal infection. Serum neutralizing antibody titers were tested at 0, 7, 14, and 28 dpi **(J)**. Significant differences are indicated with asterisks (*p < 0.05, **p < 0.01).

To evaluate the pathogenicity of this study's three BVDV genotype isolates, we performed experimental infection with healthy calves that were negative for BVDV and other common enteric and respiratory pathogens. All animals infected with BVDV developed clinical symptoms associated with varying degrees of BVDV infection, including depression, fever, conjunctivitis, nasal discharge, coughing, and diarrhea. Compared with the calves of the control group, calves infected with BVDV showed high rectal temperatures, reaching peak rectal temperatures at 7–8 dpi (Figure 8A). Only three animals infected with BVDV-1m HB-01 strain presented obviously biphasic pyrexia, with the first peak from 4 to 5 dpi (39.5°C); while the other peak was observed from 7 to 8 dpi with a maximum record of 40.7°C. The highest rectal temperatures were noticed in the calves infected with the BVDV-1m HB-01strain, followed by the calves infected with the BVDV-1a HN-03 and BVDV-1v HB-03 strains. The clinical signs' scores of the animals in each infected group reached the maximum at 7 dpi. And the clinical signs' scores of the animals infected with the BVDV-1v HB-03 strain were lower than those of calves infected with the BVDV-1a HN-03 and BVDV-1m HB-01 strains (Figure 8B). There were significant differences in daily weight gain between calves of the control group and calves infected with BVDV-1a HN-03 (*p* < 0.01), BVDV-1m HB-01 (*p* < 0.01), and BVDV-1v HB-03 (*p* < 0.05), respectively (Figure 8C). In addition, as shown in Figure 8D, one calf infected with the BVDV-1a HN-03 strain died naturally at 21 dpi. Taken together, the above results indicate that the clinical signs of calves infected with BVDV-1a HN-03 and BVDV-1m HB-01 strains were more severe than those calves infected with the BVDV-1v HB-03 strain.

#### Hematological analyses

The mean hematology counts 3 days before infection were calculated as the baseline at 0 dpi. The WBC and lymphocyte (LYM) count of all calves infected with BVDV showed a significant decrease (*p* < 0.0001) from 1 to 9 dpi compared with that of negative control calves (Figures 8E,F). The WBC count increased toward baseline levels from 7 dpi as the disease progressed (Figure 8E). All calves infected with BVDV exhibited transient leukopenia during the study based on the mean total leukocyte counts. Calves infected with BVDV-1m HB-01 strain showed a more marked response compared to calves infected with BVDV-1a HN-03 and BVDV-1v HB-03 strains, with levels dropping to more than 40% on two consecutive days (Supplementary Table 6). This is one of the characteristics of BVDV's high virulence in cattle (36). While only one calf of the BVDV-1a HN-03 and BVDV-1v HB-03 groups showed decreases in WBC counts by more than 40%, the remaining two calves showed a drop in WBC count ranging between 20 and 40% (Supplementary Table 6), which is one of the characteristics of BVDV's moderate virulence to cattle (36).

The decreasing LYM counts' trend resembled that of WBC (Figure 8F). Briefly, the LYM of all calves infected with BVDV was reduced to a different extent in the early stage of infection. As the disease progressed, the counts of LYM gradually returned to the pre-infection level from 7 dpi. Calves infected with the BVDV-1m HB-01 strain showed a slightly marked response compared to calves infected with the other two strains.

#### Viremia and shedding

To assess viremia and viral shedding, we collected nasal swabs (NSs), rectal swabs (RSs), and WBC obtained from EDTA blood samples of all calves during infection every other day and isolated viruses from these samples. Viruses were considered to be isolated from infected calves when the viruses were isolated from at least one sample each day. The virus was isolated from 1 to 14 dpi in calves infected with BVDV-1a HN-03 strain, from 1 to 11 dpi in those infected with BVDV-1m HB-01 strain, and from 1 to 9 dpi in those infected with BVDV-1v HB-03 strain, respectively (Table 2). This indicated that the shedding period of BVDV-1v HB-03 was shorter than those of BVDV-1m HB-01 and BVDV-1a HN-03 strains, respectively. In addition, viremia was demonstrated on all infected calves (Table 2). The viremia period in calves infected with BVDV-1m HB-01 strain (from 3 to 9 dpi) was longer than those in calves infected with BVDV-1a HN-03 strain (from 5 to 9 dpi) and BVDV-1v HB-03 strain (from 5 to 9 dpi). Generally, the virus was isolated more frequently from WBC than from NSs and RSs.

**Table 2:** Viral isolation from nasal swabs (NSs), rectal swabs (RSs), and white blood cells (WBC) at different time points.

**BVDV strains**	**Sample**	**No. of calves from which virus was isolated/total**						
		**no. of animals tested**						
		**1 dpi**	**3 dpi**	**5 dpi**	**7 dpi**	**9 dpi**	**11 dpi**	**14 dpi**
BVDV-1a HN-03	NSs	0/3	1/3	0/3	0/3	1/3	0/3	0/3
	RSs	1/3	0/3	1/3	2/3	2/3	0/3	1/3
	WBC	0/3	0/3	1/3	2/3	1/3	0/3	0/3
BVDV-1m HB-01	NSs	0/3	0/3	1/3	0/3	1/3	0/3	0/3
	RSs	1/3	0/3	0/3	2/3	0/3	0/3	0/3
	WBC	0/3	1/3	1/3	3/3	2/3	1/3	0/3
BVDV-1v HB-03	NSs	0/3	1/3	0/3	0/3	0/3	0/3	0/3
	RSs	1/3	0/3	0/3	2/3	1/3	0/3	0/3
	WBC	0/3	0/3	1/3	2/3	1/3	0/3	0/3
Negative control	NSs	0/3	0/3	0/3	0/3	0/3	0/3	0/3
	ASs	0/3	0/3	0/3	0/3	0/3	0/3	0/3
	WBC	0/3	0/3	0/3	0/3	0/3	0/3	0/3

Relative quantification of virus load in NSs, RSs, and bloods collected from three calves of each group at 1, 3, 5, 7, 9, and 11 dpi provided insights into the viral load of the virus following intranasal infection. Viral RNAs were detected in blood from 1 to 11 dpi in all calves infected with BVDV-1a HN-03, BVDV-1m HB-01, and BVDV-1v HB-03 strains, with a maximum number of viral RNA copies at 7, 3, and 3 dpi, respectively (Figure 8G). Viral RNAs were detected in NSs samples in all animals infected with BVDV-1a HN-03, BVDV-1m HB-01, and BVDV-1v HB-03 strains with a peak value at 3, 3, and 1 dpi, respectively (Figure 8H). Animals infected with BVDV-1a HN-03, BVDv-1m HB-01, and BVDV-1v HB-03 strains had positive shedding in anal swabs for 11 days (1–11 dpi), and the mean maximum viral RNA load (exceeded 10^7^ copies/mL) at 3, 1, and 1 dpi, respectively (Figure 8I). Generally, the mean viral RNA load in samples from animals infected with BVDV strains was the highest in the BVDV-1m HB-01 group, followed by the BVDV-1a HN-03 group and the lowest in the BVDV-1v HB-03 group.

#### Systemic distribution of viral RNA

To assess the distribution of BVDV in inoculated calves, viral RNAs were determined by RT-PCR in different tissues from euthanized calves at 28 dpi. The virus was still detected in multiple tissues of calves euthanized at the end of the experiment, at 28 dpi, when viremia of the infected calves disappeared. BVDV was detected in different tissues from two calves infected with BVDV-1a HN-03, two calves infected with BVDV-1m HB-01, and one calf infected with BVDV-1v HB-03 (Table 3). BVDV was most frequently detected in (i) lymphoid tissues, (ii) lungs, (iii) tonsils, (iV) spleens, and (v) intestinal tract (jejunums and ileums). In addition, tissues like hearts, livers, and kidneys were not found to harbor the virus.

**Table 3:** Viral RNAs were determined by reverse transcription polymerase chain reaction (RT-PCR) in different tissues from euthanized calves at 28 dpi.

**Tissues**	**No. of calves tested positive for viral**			
	**RNAs/total no. of calves tested**			
	**BVDV-1a** **HN-03^a^**	**BVDV-1m** **HB-01**	**BVDV-1v** **HB-03**	**Negative** **control**
Heart	0/3	0/3	0/3	0/3
Lung	2/3^b^	2/3	1/3	0/3
Liver	0/3	0/3	0/3	0/3
Kidney	0/3	0/3	0/3	0/3
Spleen	1/3^b^	1/3	0/3	0/3
Duodenum	0/3	0/3	0/3	0/3
Jejunum	1/3^b^	0/3	0/3	0/3
Ileum	1/3^b^	1/3	0/3	0/3
Tonsil	2/3^b^	1/3	1/3	0/3
Superficial cervical	2/3^b^	2/3	1/3	0/3
lymph node				
Mesenteric	1/3	1/3	0/3	0/3
lymph node				
Blood	0/3	0/3	0/3	0/3

a Means one calf died naturally at 21dpi.

b Means the sample of calf died at 21 dpi was tested positive.

#### Histopathology

**Figure 9:**
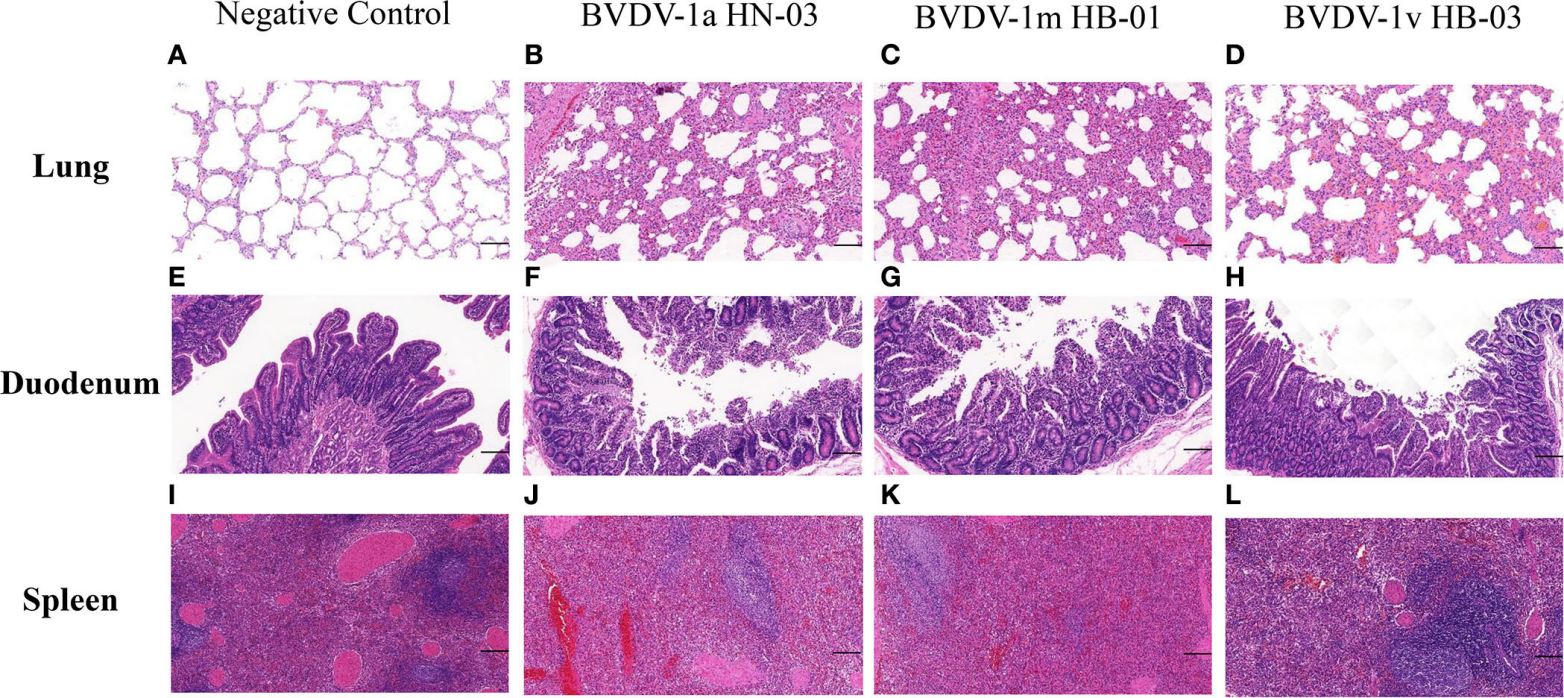
Histopathological changes of lung, duodenum, and spleen. **(A–L)** Represent groups of the negative control, BVDV-1a HN-03, BVDV-1m HB-01, and BVDV-1v HB-03, respectively. Compared with the normal tissue structure **(A)**, the alveolar structure of calves infected with bovine viral diarrhea virus (BVDV) was destroyed **(B–D)**. Compared with the normal duodenal tissues **(E)**, the duodenum of the calves infected with bovine viral diarrhea virus (BVDV) **(F–H)** had a large amount of small intestinal chorion shed in the intestine. Compared with the normal spleen tissues **(I)**, the spleen of the calves infected with BVDV **(J,K)** showed a significantly wide periarterial lymphatic sheath, with increased bleeding points and lymphocytes depletion in red pulp. The horizontal line on the bottom right of each figure is the scale bar (100 μm scale).

The main histopathological changes were observed in the lungs, digestive tract, and spleens. These changes included the destruction of the normal pulmonary alveoli architecture, a large amount of small intestinal chorion shed in the intestine, and increased bleeding points in the spleen in all the infected calves (Figure 9). None of the tissue samples from the negative control calves showed pathological changes. The degree of histopathological changes of the calves infected with BVDV-1a HN-03 and 1m HB-01 strains was more serious than that of calves infected with BVDV-1v HB-03 strain.

#### Neutralizing antibody titers and antigenicity

Neutralizing antibodies against each homologous strain were assessed in serum samples obtained at 0, 7, 14, 21, and 28 dpi (Figure 8J). Calves infected with BVDV had been detected with neutralizing antibodies from 7 to 28 dpi, and the mean neutralizing antibody titers increased as the disease progressed, until the highest values were reached at 28 dpi. The mean neutralizing antibody titers of calves infected with BVDV-1v HB-03 were significantly lower (*p* < 0.01) than those of calves infected with BVDV-1a HN-03 and 1m HB-01 at 14 dpi. At each time point, the mean neutralizing antibody titer was the highest in calves infected with BVDV-1m HB-01, followed by calves infected with BVDV-1a HN-03, and that was the lowest in calves infected with BVDV-1v HB-03.

To determine the cross-neutralizing antibody titers among the BVDV genotypes in the current study, the serum samples collected from calves infected with BVDV at 28 dpi were used to perform the cross-neutralization experiment. The highest titers were observed in the homologous pairs compared to the viruses from different genotypes (Table 4). Except for the control groups, the serum from all the infected groups neutralized all the viruses, with some remarkable differences in the neutralization titers. The antiserum developed with the BVDV-1v HB-03 strain showed a high neutralization actively (>1:80) against the BVDV-1a, BVDV-1b, and BVDV-1m strains. Based on the cross-neutralization titers, the levels of the serological relatedness among the studied genotypes were calculated and exhibited by the *R*-values (Table 5). The low *R*-values (<25) were observed among the BVDV-1a HN-03, BVDV-1m HB-01, and BVDV-1v HB-03 strains, indicating low antigenic similarity within these genotypes.

**Table 4:** Cross-neutralization titers of antisera against different strains of isolated bovine viral diarrhea virus (BVDV) genotypes.

**Viruses**	**Neutralization titers of antisera**			**Control**
	**against these BVDVs**			**group**
	**BVDV-1a** **HN-03**	**BVDV-1m** **HB-01**	**BVDV-1v** **HB-03**	
BVDV-1a HN-03	526	58	80	<1:5
BVDV-1m HB-01	58	813	102	<1:5
BVDV-1v HB-03	80	20	406	<1:5
BVDV-1b AV69	66	14	94	<1:5

**Table 5:** Serological relatedness expressed as coefficient of antigenic similarity (*R*) values of BVDV-1 genotypes.

	**BVDV-1a HN-03**	**BVDV-1m** **HB-01**	**BVDV-1v** **HB-03**
BVDV-1a HN-03	100	8.9	17.3
BVDV-1m HB-01	8.9	100	7.9
BVDV-1v HB-03	17.3	7.9	100
BVDV-1b AV69	NA	NA	NA

NA, No BVDV-1b antiserums.

## Discussion

### Novel genotype BVDV-1v HB-03 isolation

As an important viral pathogen, BVDV endangers cattle health and the cattle industry economy (37). Previous studies have summarized the BVDV genotypes identified in China over the past years, including 11 known genotypes (1a, 1b, 1c, 1d, 1m, 1o, 1p, 1q, 1u, 2a, and 2b) (11, 38) and two novel genotypes 1v (15, 16) and 1w (15). Even the most recent study revealed the first evidence of HoBi-like pestivirus infection in Chinese cattle herds (39). However, most research focuses on nucleic acid detection with the 5′-UTR regions for viral identification and classification due to the difficulties in isolating the virus, resulting in the lack of reliable evidence for the genotyping and comprehensive genetic characterization analysis of BVDV (40). One of the examples is the identification of the emerging genotypes BVDV-1v in China. Initially, Deng et al. reported that the novel genotype BVDV-1v was based on 5′-UTR and Npro sequences (15). In a subsequent study, nine BVDV isolates were identified as BVDV-1v based on the 5′-UTR and BVDV-1o based on N^pro^ (17). Tian et al. revealed that these and other recently isolated strains were designated as BVDV-1v (16). Furthermore, some strains cannot be further verified as BVDV-1v due to a lack of N^pro^ sequence information (17). In this study, we isolated the BVDV-1v HB-03 strain from a calf with severe diarrhea and confirmed the novel genotype BVDV-1v based on the phylogenetic analysis of multiregion sequences (including 5′-UTR, N^pro^, E2, and complete genome), indicating the reliability in identifying the novel genotype BVDV-1v. In addition, phylogenetic analysis of the other six segments showed that the BVDV-1v HB-03 strain formed a single clade, illustrating its genetic divergence from the established and proposed BVDV genotype.

Several studies have reported the genetic diversity of BVDV, and 1m, 1b, 1a, and 1c were the dominant genotypes in China (11, 12, 15, 41, 42). While the novel genotype 1v has recently been reported in dairy herds in Heilongjiang, Shandong, and Inner Mongolia (15), suggesting that the novel virus has spread to cattle herds in China. A reasonable explanation for the wide spread of the disease among cattle herds is attributed to the rapid development of the cattle industry and the frequent cattle transportation from various places in China.

### Recombination analysis of the novel genotype BVDV-1v

The pestivirus gene recombination plays an important role in the evolution of viruses, as it changes the adaptation of viruses to hosts, the virulence *in vitro* and *in vivo*, and in the emergence of new viruses (43). In this study, to identify significant recombination events in the BVDV-1v HB-03 strain genome, the recombination evidence was determined by at least four methods using the RDP 4 program coupled with a similarity plotting. The BVDV-1v HB-03 strain was observed to have two obvious recombination signals, and their parental strains were identified as BVDV-1m (LN-1 strain isolated from cattle in northern China in 2014, GenBank Accession Number: KT896495) and BVDV-1q [SD0803 strain isolated from swine in northern China in 2008 (44)], BVDV-1o [IS26/01ncp strain isolated from cattle in Japan in 2001 (45)], and BVDV-1m [SD-15 strain isolated from cattle in northern China in 2015 (23)], respectively. Recombination occurs when at least two viruses co-infect the same host cell and exchange their genetic segments (46). Therefore, recombination events of the BVDV-1v HB-03 strain hinted at the occurrence of cross-genotype and cross-geographical region transmission events, which could be explained as follows. Most of the cattle were transported from the northern China where the cattle industry was developed, to southern and central China, where the beef and dairy products were in high demand, such as the BVDV-1v HB-03 strain isolation origin, Hubei province. Therefore, we speculate that frequent trade and herd transportation might promote the disease's spread, increase the virus's genetic diversity, and generate novel genotypes (like BVDV-1v). In addition, cattle were the natural host of BVDV and are considered the main source of BVDV infection for swine (47). When cattle and pigs are raised on the same farm, the pig herd will be infected with BVDV (48). Although industrial domesticated animal production facilities have increased rapidly, it is common for multiple domestic animals to be raised on the same farm in China (49). Additionally, BVDV was detected in feces and in other specimens of domestic and wild ruminants at the wildlife–livestock interface, suggesting that direct and/or indirect contact with other ruminants and wildlife can result in an efficient BVDV dissemination (50–52). Using contaminated biological products, such as semen or embryo transfer, also promoted the transmission of BVDV (53). Over the past decades, China had continuously imported Holstein semen and embryos, mainly from North America and Europe (54). China has become the world's largest importer of animal products, including imported live cattle (55). Therefore, we speculate that direct and/or indirect contact between these animals might lead to cross-genotype transmission of BVDV and produce recombinant viruses.

### E2 protein mutation of the novel genotype BVDV-1v

The E2 protein is involved in attachment and entry into target cells (56). As the potential host cell binding site, the 141–170 amino acids of pestivirus E2 help the virus enter the host (57). In addition, cellular β-actin interacted with pestivirus E2 (182–261 and 262–341 aa) and promoted virus entry and the endocytosis (34). This study shows the variation of these amino acids in BVDV-1v HB-03 strain E2 (I159, S195, A204, N229, W236, A252, and T272) may change the viral characteristics, such as reduced replication ability and virulence. In addition, previous studies have shown that the E2 antigenic domains DA and DB (4–87 and 88–164 aa, respectively), are likely to be most exposed on the surface of the virus and responsible for antigen specificity (34, 35, 58). And the neutralizing antigenic 64–76 aa in E2 of pestivirus was indispensable for reserving the structural integrity of epitopes (59, 60). BVDV-1v HB-03 strain E2 has a variation at position Y65F compared with the representative strain of each BVDV-1 genotype, indicating possible changes in the antigenicity in the BVDV-1v HB-03 strain. Furthermore, *in vivo* infection of BVDV suggested that the pathogenicity and neutralizing antibody titers of the BVDV-1v HB-03 strain were lower than those of BVDV-1a HN-03 and BVDV-1m HB-01 strains. Determining the associations between E2 mutations and viral characteristics (pathogenicity and antigenicity) will be addressed in future research. Previous studies have indicated that the antigenic variation of E2 among *Pestiviruses* is crucial to cross-neutralization, which may lead to incomplete E2 vaccine protection concerning heterologous strains (34).

### Viral replication and pathogenicity

One-step growth curves can assess the level of viral replication. The growth trends of the three BVDV isolates were semblable, which are similar to those of the previous studies (61, 62). During replication, the virus titer level of the BVDV-1v HB-03 strain was significantly lower than those of the BVDV-1a HN-03 strain and BVDV-1m HB-01, suggesting the low replication level of the BVDV-1v HB-03 strain. The TCID_50_ level of BVDV-1v HB-03 strain at 0 hpi was the lowest among the three isolates in this study, suggesting that the low adsorption capacity of the BVDV-1v HB-03 strain resulted in its low replication level. Considering the important functions of E2 protein in viral entry and endocytosis discussed above, we speculate that the mutation in the E2 protein of BVDV-1v HB-03 strain may be the molecular mechanism of its low adsorption level. Mutations in other genomic regions can be involved in a low replication rate. A previous study showed that a mutation in BVDV NS2 attenuates viral RNA replication (63).

BVDV causes immunosuppressive effects in calves after naturally occurring infection, either transient or persistent (64), resulting in secondary infections of various pathogens. And in clinical cases, BVDV often exacerbates the progression of disease and severity of pathology in the form of co-infection with other pathogens (18). In this study, the animals we selected for experimental infection were not only identified as negative for BVDV antigen and antibody but also determined to be free of infection with several other common pathogens. The clear background of the infected calves ensured the reliability of the evaluation of the pathogenicity of the three BVDV isolates in this study.

Calves infected with high and low virulent BVDV strains show leukopenia (20, 36, 65–67). The decrease in leukopenia is slightly different, indicating that the BVDV-1m HB-01 strain makes it easier for infected animals to acquire secondary infections than the low-virulence strains. However, the interesting observation is that the decrease and reversion trend of WBC and LYM counts are comparable, which suggests that the value by itself may not be a characteristic strictly related to the virulence of the three BVDV isolates in this study. Therefore, as described below, it is important to consider other features, such as clinical signs, viremia and shedding, and histopathological changes.

We observed obviously biphasic pyrexia only in calves infected with the BVDV-1m HB-01 strain. This clinical sign of infected calves has been reported in multiple previous studies (4, 20, 68). The severity of pyrexia after BVDV infection is related to the virulence of the strain, suggesting the high virulence of the BVDV-1m HB-1 strain. The calves of the three groups all developed gastrointestinal syndrome and respiratory signs after infection, and the severity and duration of the signs were different. Compared with the other two isolates, the BVDV-1v HB-03 strain resulted in mild clinical symptoms for calves, which was observed in calves infected with other moderately virulent strains (36).

The severity of clinical symptoms may be related to the level of viremia and viral shedding (20, 32). In this study, all infected calves could detect the virus from NSs and RSs, indicating that acutely infected animals are also transmitters of the virus. The virus was more frequently isolated from WBC than from NSs and RSs, which further supports the conclusion that WBC samples are more suitable for determining the presence of the virus during acute infection (69). The peak amounts of BVDV RNA in blood, NSs, and RSs were comparable and consistent with those of a previous study (20). However, the calves infected with the three BVDV isolates in this study showed differences in the time span of viremia (virus isolated from WBC) and shedding (virus isolated from NSs and RSs), indicating the significantly longer period of viral excretion of the high-virulence strain BVDV-1m HB-01 strain.

The virus was still detected mainly in the immune-related tissue (lymphoid tissues and tonsils) of calves euthanized at the end of the experiment (28 dpi) when viremia of the infected calves disappeared. And histopathological changes were observed mostly in these organs and tissues. Several studies have reported that lymphoid tissues play an important role in virus replication (20, 69), and tonsils are among the earliest viral replication sites (4). Our results demonstrated the immunosuppression triggered by BVDV infection, which explains the susceptibility of BVDV-infected cattle to opportunistic pathogens. In addition, we observed varying degrees of respiratory symptoms, pathological damage in the lungs, and virus detection from the lungs. These results provide further direct experimental evidence that BVDV contributes to the bovine respiratory disease complex (BRDC) (70, 71). These results also show that these effects (immunosuppression and BRDC) increase with the virulence of the isolates (BVDV-1v HB-03 < BVDV-1a HN-03 < BVDV-1m HB-01). Considering the available data, BVDV-1m HB-01 was identified as a highly virulent strain, and BVDV-1a HN-03 and BVDV-1v HB-03 were both identified as moderately virulent strains.

### Serological relationships among genotypes

The antigenic relationship of BVDV isolates plays an important role in diagnosis and immunization strategies. The cross-neutralization test has been widely used to determine the similarity and difference of antigens (21, 22, 72). Although the antigenic differences among genotypes of BVDV-1 have been reported (21), the antigenic relationships among the dominant genotypes 1m in China, novel 1v and other BVDV-1 strains are not yet clear. In this study, most of the cross-neutralizing antibody titers among BVDV-1 genotypes (1a, 1b, 1m, and 1v) were <1:100. These antibody titers are below the recommended cut-off protection value (73, 74). The antigenic similarity coefficient (*R*) value is defined as the serological relationship between genotypes, and *R*-value of <25 indicates significant antigenic differences (75). In this study, the *R*-values among Chinese genotypes strongly suggested their antigenic diversity, which is completely different from the high similarity (*R* > 25) among BVDV-1 genotypes (1a, 1b, 1h, 1k, and 1e) in previous studies (76). Only BVDV-1a-inactivated vaccines have been used to prevent BVDV in China. It is important to consider that the presence of multiple genotypes and antigenic diversity of BVDV isolates may affect the protective efficacy of BVDV vaccines. A vaccination may fail to protect against virus infection due to the genetic and antigenic variability among different strains (77). BVDV-1b and BVDV-1m are the predominant genotypes in China (11). Therefore, it is imperative to evaluate comprehensively the cross-protection between the existing inactivated vaccines (BVDV-1a) and the current superior, dominant genotypes in China.

## Conclusion

In this study, three BVDV isolates (BVDV-1a HN-03, BVDV-1m HB-01, and BVDV-1v HB-03) were isolated from calves with severe diarrhea in China and identified as BVDV-1a, 1m, and novel genotype 1v, respectively, based on the phylogenetic analysis of multiple genomic regions and nearly complete genome. The novel genotype BVDV-1v HB-03 strain showed multiple genetic variation sites related to virus replication and antigenic epitopes. And recombination analysis of BVDV-1v HB-03 strain hinted at the possible occurrence of cross-genotype and cross-geographical region transmission events. Based on assessing different parameters *in vitro* and *in vivo*, BVDV-1m HB-01 was identified as a highly virulent strain, and BVDV-1a HN-03 and BVDV-1v HB-03 were both identified as moderately virulent strains. The cross-neutralization test demonstrated the antigenic diversity among these Chinese genotypes (1a, 1m, and 1v).

Our findings illustrate the genetic evolution characteristics of the emerging genotype and the pathogenic mechanism of different genotype strains, and identify potential vaccine candidate strains (BVDV-1m HB-01 and BVDV-1a HN-03) and suitable BVDV challenge strain (BVDV-1m HB-01) for the comprehensive prevention and control of BVDV. In particular, for the epidemiological situation in China, more BVDV genotype strains should be considered in the vaccine formulation of BVD.

## Data availability statement

The datasets presented in this study can be found in online repositories. The names of the repository/repositories and accession number(s) can be found in the article/Supplementary material.

## Ethics statement

The animal study was reviewed and approved by the protocol (HZAUCA-2018-005) regarding animal infection was approved by the Committee on the Ethics of Animal Experiments at Huazhong Agricultural University and conducted in strict accordance with the Guide for the Care and Use of Laboratory Animals, Hubei Province, China. Written informed consent was obtained from the owners for the participation of their animals in this study.

## Author contributions

AG and JZ: conceptualization. JZ and CW: data curation. JZ: formal analysis and writing—original draft. AG: funding acquisition, resources, project administration, and writing—review and editing. JZ, CW, LZ, TZ, HL, YW, KX, MQ, and QP: methodology. YC, CH, XC, and JC: interpretation and discussion of the results. HC and AG: supervision. All authors contributed to the article and approved the submitted version.

## Funding

This work was supported by the Key Research and Development Program of the Ningxia Hui Autonomous Region (#2021BEF02028), Hubei Provincial Key Research and Development Program of China (2020BBA055), and Chinese Agricultural Research System of MOF and MARA (CARS-37).

## Conflict of interest

Author QP was employed by the company Wuhan Keqian Biology Co. Ltd., Wuhan, China. The remaining authors declare that the research was conducted in the absence of any commercial or financial relationships that could be construed as a potential conflict of interest.

## Publisher's note

All claims expressed in this article are solely those of the authors and do not necessarily represent those of their affiliated organizations, or those of the publisher, the editors and the reviewers. Any product that may be evaluated in this article, or claim that may be made by its manufacturer, is not guaranteed or endorsed by the publisher.
