# Supplementary material for: Isolation of BVDV-1a, 1m, and 1v strains from diarrheal calf in china and identification of its genome sequence and cattle virulence
Source: Front Vet Sci. 2022 Nov 16;9:1008107. doi: 10.3389/fvets.2022.1008107 (PMC9709263; doi:10.3389/fvets.2022.1008107)
Supplement: Supplementary file 1 [file Table_1.DOCX]

**Supplementary materials**

**Table S1** Primers used for amplification of the complement genome sequence of BVDV-1v HB-01

| Primers | Primer sequence (5’-3’) | Location in genome (nt) |
| --- | --- | --- |
| Gap-F: | ATGGTTGAGGTTTGGTGC | 2476-2493 |
| Gap-R | CTGGTCCCTCGCTTTGTA | 3200-3217 |

**Table S2** Reference pestiviruses used for sequence and phylogenetic analyses

| Pestivirus pecies | Subtype | Strain | Origin | Location | Collection year | GenBank accession number | 5-UTR | Npro |
| --- | --- | --- | --- | --- | --- | --- | --- | --- |
| BVDV-1 | 1a | NADL | Cattle | USA | 1963 | AJ133739 | - | - |
| BVDV-1 | 1a | Singer_Arg | - | Argentina | 2007 | DQ088995 | - | - |
| BVDV-1 | 1b | CC13B | Cattle | China | 2013 | KF772785 |  |  |
| BVDV-1 | 1b | Av69 VEDEVAC | Cattle | USA | 2011 | KC695814 |  |  |
| BVDV-1 | 1b | VEDEVAC | Cattle | Hungary | 2003 | AJ585412 | - | - |
| BVDV-1 | 1b | Osloss | Cattle | Germany | 1965 | M96687 | - | - |
| BVDV-1 | 1b | JL-1 | Cattle | China | 2009 | KF501393 |  |  |
| BVDV-1 | 1c | Bega-like | Bovine | Australia | 2012 | KF896608 | - | - |
| BVDV-1 | 1d | 10JJ-SKR | Cattle | South Korea | 2010 | KC757383 | - | - |
| BVDV-1 | 1e | Carlito | Cattle | Switzerland | 2014 | KP313732 | - | - |
| BVDV-1 | 1f | J-Au | Cattle | Austria | 1998 | - | AF298067 | AF287286 |
| BVDV-1 | 1f | W-Au | Cattle | Austria | 1998 | - | AF298073 | AF287290 |
| BVDV-1 | 1f | SLO/136/2014 | Cattle | Slovenia | 2014 | MH899943 | MH899943 | MH899943 |
| BVDV-1 | 1g | A-Au | Cattle | Austria | 1998 | - | AF298064 | AF287283 |
| BVDV-1 | 1g | L-Au | Cattle | Austria | 1998 | - | AF298069 | AF287287 |
| BVDV-1 | 1h | G-Au | Cattle | Austria | 1998 | - | AF298066 | AF287285 |
| BVDV-1 | 1h | UM/126/07 | Cattle | Italy | 2016 | LT631725 | - | - |
| BVDV-1 | 1i | 23-15 | Cattle | UK | 1997 | - | AF298059 | AF287279 |
| BVDV-1 | 1i | ACM/BR/2016 | Cattle | Brazil | 2017 | KX857724 | - | - |
| BVDV-1 | 1j | KS86-1ncp | Cattle | Japan | 2002 | AB078950 | - | - |
| BVDV-1 | 1j | KS86-1cp | Cattle | Japan | 2002 | AB078952 | - | - |
| BVDV-1 | 1k | SuwaCp | Cattle | Switzerland | 1999 | KC853441 | AF117699 | AY894998 |
| BVDV-1 | 1k | Suwancp | Cattle | Switzerland | 2000 | KC853440 | - | - |
| BVDV-1 | 1l | 71-15 | Cattle | France | 2008 | - | KF205306 | KF205329 |
| BVDV-1 | 1l | 71-16 | Cattle | France | 2008 | - | KF205307 | KF205330 |
| BVDV-1 | 1m | SD-15 | Cattle | China | 2015 | KR866116 | - | - |
| BVDV-1 | 1m | ZM-95 | Pig | China | 1995 | AF526381 | - | - |
| BVDV-1 | 1m | XC | Cattle | China | 2015 | MH166806 |  |  |
| BVDV-1 | 1m | LN-1 | Cattle | China | 2014 | KT896495 | - | - |
| BVDV-1 | 1n | Shitara/02/06 | Cattle | Japan | 2006 | LC089876 | - | - |
| BVDV-1 | 1n | So CP/75 | Cattle | Japan | 1975 | - | AB359929 | AB359929 |
| BVDV-1 | 1o | IS26/01ncp | Cattle | Japan | 2001 | LC089875 | - | - |
| BVDV-1 | 1o | IS25CP/01 | Cattle | Japan | 2001 | - | AB359931 | AB359931 |
| BVDV-1 | 1o | AQGN96BI5 | Cattle | China | 1996 | - | AB300691 | - |
| BVDV-1 | 1p | BJ0701 | Cattle | China | 2007 | - | GU120247 | GU120259 |
| BVDV-1 | 1p | BJ0702 | Cattle | China | 2007 | - | GU120248 | GU120260 |
| BVDV-1 | 1p | BJ0703 | Cattle | China | 2007 | - | GU120249 | GU120261 |
| BVDV-1 | 1q | SD0803 | Pig | China | 2008 | JN400273 | - | - |
| BVDV-1 | 1q | Camel-6 | Camel | China | 2010 | KC695810 | - | - |
| BVDV-1 | 1q | 11N36 | Cattle | China | 2011 | - | JX437156 | - |
| BVDV-1 | 1r | VE/245/12 | - | Italy | - | - | LM994671 | LN515610 |
| BVDV-1 | 1r | CA/181/10 | - | Italy | - | - | LM994672 | LN515609 |
| BVDV-1 | 1r | Monopartite | Unknown | Italy | 2017 | LT797813 | LT797813 | LT797813 |
| BVDV-1 | 1s | UM/136/08 | Cattle | Italy | 2011 | - | LM994673 | LN515612 |
| BVDV-1 | 1t | SI/207/12 | Cattle | Italy | 2007 | - | LM994674 | LN515611 |
| BVDV-1 | 1u | M31182 | Yak | China | 2010 | JQ799141 | - | - |
| BVDV-2 | 2a | HLJ-10 | Cattle | China | 2011 | JF714967 | - | -- |
| BVDV-2 | 2a | JZ05-1 | Cattle | China | 2005 | GQ888686 | - | - |
| BVDV-2 | 2a | XJ-04 | Cattle | China | 2004 | FJ527854 | - | - |
| BVDV-2 | 2b | Hokudai-Lab/09 | Bovine | Japan | 2010 | AB567658 | - | - |
| BVDV-2 | 2b | SD1301 | Cattle | China | 2012 | KJ000672 | - | - |
| BVDV-2 | 2c | NRW19-13-8_Dup | Cattle | Germany | 2013 | HG426483 | - | - |
| BVDV-2 | 2c | Potsdam 1600 | Cattle | Germany | 2000 | HG426491 | - | - |
| BVDV-2 | 2c | SH2210-23 | Cattle | Germany | 2010 | HG426494 | - | - |
| BDV | BDV | D31 | Lamb | USA | - | U70263 | - | - |
| BDV | BDV | X818 | Sheep | Germany | - | AF037405 | NC_003679 | NC_003679 |
| CSFV | CSFV | cF114 | - | China | - | AF333000 | - | - |

**Table S3** Clinical signs and designated score

|  | Score | Clinical signs |
| --- | --- | --- |
| Conjunctivitis | 0 | None (Normal condition, pale pink conjunctiva with no sign of the blood vessels) |
|  | 1 | Slight (Slight hyperaemia of the conjunctiva) |
|  | 2 | Moderate (Swollen hyperaemic conjunctiva, hyperaemic, reddish conjunctiva , slight discharge accumulating in the nasal canthus of the eye) |
|  | 3 | Severe (Swollen dark red conjunctiva, thick discharge, eyelashes sticky with discharge in a large part of the palpebral aperture) |
| Nasal discharge | 0 | None (Normal condition, no discharge) |
|  | 1 | Slight (Slight serous discharge confined to nare, remarkable serous discharge dripping from muzzle) |
|  | 2 | Moderate (Slight catarrhal (mucous) discharge, thick catarrhal(mucous) discharge) |
|  | 3 | Severe (Yellow-green(mucopurulent) discharge) |
| Coughing | 0 | None (No coughing) |
|  | 1 | Slight (1-2 independent coughs, now and then) |
|  | 2 | Moderate (Frequent coughing) |
|  | 3 | Severe (Frequent or continuous painful, convulsive coughing) |
| Abnormal breathing | 0 | None (Normal breathing) |
|  | 1 | Slight (Shortness of breath) |
|  | 2 | Moderate (Continuous shortness of breath, sometimes definite labored breathing) |
|  | 3 | Severe (Continuous shortness of breath, frequently definite laboured breathing or continuous laboured breathing) |
| Diarrhoea | 0 | None (Normal stool (defecation frequency, colour, quantity, consistency)) |
|  | 1 | Slight (Looser than usual stool, normal colour, quantity and defecation frequency or losse stool, elevated defecation frequency) |
|  | 2 | Moderate (Very loose, unformed stool, elevated defecation frequency) |
|  | 3 | Severe (Watery profuse diarrhea, spasms, pain, bloody or blood-tinged stool) |
| Appetite | 0 | Normal (Eager to be fed at the regular feeding time) |
|  | 1 | Reduced (Less eager in at least one of the feeding times) |
|  | 2 | Strongly reduced |
|  | 3 | Anorexia (Loss of appetite) |

**Table S4** Complete genomic information and coding potential of the BVDV HB-03 strain

| BVDV-1a HN-03 | | | | | |
| --- | --- | --- | --- | --- | --- |
| Region | Location(nt) | G+C% | Length (nt) | Protein | Length (aa) |
| ORF | 383-12,079 |  | 11,697 |  | 3898 |
| N^pro^ | 383-886 | 45 | 504 | p20 | 168 |
| Capsid | 887-1192 | 43 | 306 | p14 | 102 |
| Erns | 1193-1873 | 46 | 681 | pgp48 | 227 |
| E1 | 1874-2458 | 45 | 585 | gp25 | 195 |
| E2 | 2459-3580 | 45 | 1122 | gp53 | 374 |
| P7 | 3581-3790 | 46 | 210 | p7 | 70 |
| NS2-3 | 3791-7198 | 46 | 3408 | p125 | 1136 |
| NS4A | 7199-7390 | 48 | 192 | p10 | 64 |
| NS4B | 7391-8431 | 43 | 1041 | p32 | 347 |
| NS5A | 8432-9919 | 46 | 1488 | p58 | 496 |
| NS5B | 9920-12,076 | 45 | 2157 | p75 | 719 |
| BVDV-1m HB-01 | | | | | |
| Region | Location(nt) | G+C% | Length (nt) | Protein | Length (aa) |
| ORF | 330-12,032 | 46 | 11,703 |  | 3900 |
| N^pro^ | 330-833 | 44 | 504 | p20 | 168 |
| Capsid | 834-1145 | 43 | 312 | p14 | 104 |
| Erns | 1146-1826 | 49 | 681 | pgp48 | 227 |
| E1 | 1827-2411 | 45 | 585 | gp25 | 195 |
| E2 | 2412-3533 | 48 | 1122 | gp53 | 374 |
| P7 | 3534-3743 | 42 | 210 | p7 | 70 |
| NS2-3 | 3744-7151 | 47 | 3408 | p125 | 1136 |
| NS4A | 7152-7343 | 45 | 192 | p10 | 64 |
| NS4B | 7344-8384 | 44 | 1041 | p32 | 347 |
| NS5A | 8385-9872 | 46 | 1488 | p58 | 496 |
| NS5B | 9873-12,029 | 45 | 2157 | p75 | 719 |
| BVDV-1v HB-03 | | | | | |
| Region | Location(nt) | G+C% | Length (nt) | Protein | Length (aa) |
| ORF | 268-11,966 | 46 | 11,699 |  | 3898 |
| N^pro^ | 268-771 | 46 | 504 | p20 | 168 |
| Capsid | 772-1083 | 41 | 312 | p14 | 104 |
| Erns | 1084-1764 | 49 | 681 | pgp48 | 227 |
| E1 | 1765-2349 | 46 | 585 | gp25 | 195 |
| E2 | 2350-3471 | 46 | 1122 | gp53 | 374 |
| P7 | 3472-3681 | 45 | 210 | p7 | 70 |
| NS2-3 | 3682-7089 | 47 | 3408 | p125 | 1136 |
| NS4A | 7090-7281 | 43 | 192 | p10 | 64 |
| NS4B | 7282-8322 | 44 | 1041 | p32 | 347 |
| NS5A | 8323-9810 | 47 | 1487 | p58 | 495 |
| NS5B | 9811-11,964 | 45 | 2151 | p75 | 717 |

**Table S5** The unique amino acid sequence revealed within HB-03-encoded

| Strains-Genotype | Amino acid location (aa) | | | | | | | | | | | | |
| --- | --- | --- | --- | --- | --- | --- | --- | --- | --- | --- | --- | --- | --- |
|  | DA | |  | DB |  | DC | | | | | |  | DD |
|  | 35 | 65 |  | 159 |  | 194 | 195 | 204 | 229 | 236 | 252 |  | 272 |
| Consensus | D | Y |  | S |  | Q | L | S | E | L | T |  | K |
| HB-03 (1v) | V | F |  | I |  | R | S | A | N | W | A |  | T |
| HN-03 (1a) | P | **●** |  | **●** |  | **●** | **●** | **●** | **●** | **●** | Q |  | **●** |
| Av69(1b) | **●** | **●** |  | H |  | **●** | **●** | **●** | K | Q | H |  | R |
| Bega-like (1c) | P | **●** |  | P |  | **●** | **●** | **●** | **●** | **●** | Q |  | **●** |
| 10JJ-SKR (1d) | **●** | **●** |  | G |  | **●** | **●** | **●** | K | **●** | Q |  | **●** |
| Carlito (1e) | A | **●** |  | T |  | **●** | **●** | **●** | T | **●** | N |  | **●** |
| UM/126/07 (1h) | **●** | **●** |  | T |  | **●** | T | T | K | **●** | T |  | **●** |
| ACM/BR/2016 (1i) | I | **●** |  | **●** |  | **●** | **●** | **●** | **●** | **●** | H |  | **●** |
| KS86-1ncp (1j) | P | **●** |  | **●** |  | **●** | **●** | H | **●** | **●** | **●** |  | **●** |
| Suwancp (1k) | **●** | **●** |  | **●** |  | W | **●** | **●** | K | **●** | **●** |  | **●** |
| HB-01 (1m) | **●** | **●** |  | A |  | **●** | **●** | N | V | I | Q |  | **●** |
| Shitara/02/06 (1n) | A | **●** |  | T |  | M | M | **●** | V | T | **●** |  | **●** |
| IS26/01ncp (1o) | E | **●** |  | T |  | **●** | **●** | T | K | V | H |  | **●** |
| SD0803 (1q) | **●** | **●** |  | V |  | **●** | V | T | **●** | **●** | **●** |  | **●** |
| M31182 (1u) | K | **●** |  | A |  | P | **●** | **●** | A | I | **●** |  | S |

Note: Black dots indicate consensus amino acids.

**Table S6** Percentage of decrease of the counts of WBC post virus inoculation (%)

| Group | Animals ID | Days post challenge | | | | | | |
| --- | --- | --- | --- | --- | --- | --- | --- | --- |
|  |  | 1 | 3 | 5 | 7 | 9 | 11 | 14 |
| BVDV HB-03 | 428 | -13.7 | **-42.5** | **-57.5** | **-53.4** | **-50.7** | -34.2 | **-56.2** |
|  | 501 | -13.5 | -22.5 | -22.5 | -24.7 | -18 | -4.5 | 0 |
|  | 505 | 36.4 | -23.4 | -28.0 | -34.6 | 18.7 | 1.9 | 12.1 |
| BVDV  HB-01 | 763 | -2.5 | -21.5 | -27.8 | **-43.0** | **-40.0** | -36.7 | -7.6 |
|  | 764 | -29.2 | **-43.1** | **-51.4** | -33.3 | -29.2 | -25.0 | -31.9 |
|  | 767 | -16.2 | -35.1 | **-41.9** | -4.1 | 33.1 | 70.3 | 35.1 |
| BVDV HN-03 | 774 | -20.4 | -34.4 | -6.5 | -12.9 | -17.2 | -21.5 | -31.2 |
|  | 776 | -3.6 | **-41.1** | **-51.8** | -5.4 | 0.0 | 5.4 | -14.3 |
|  | 752 | 1.6 | 3.2 | -7.9 | -17.5 | -11.1 | -4.8 | -3.2 |
| Negative Control | 415 | 1.1 | -5.5 | -8.8 | -11.0 | -14.3 | 18.3 | -38.5 |
|  | 422 | 2.8 | 0.9 | -0.9 | -10.4 | -4.7 | 0.0 | -1.9 |
|  | 507 | 9.6 | -19.2 | -16.3 | -14.4 | -17.3 | -6.7 | -13.5 |

Note: The counts of WBC decreased more than 40% were marked bold.
